# Critical Review of Health Impacts of Wildfire Smoke Exposure

**DOI:** 10.1289/ehp.1409277

**Published:** 2016-04-15

**Authors:** Colleen E. Reid, Michael Brauer, Fay H. Johnston, Michael Jerrett, John R. Balmes, Catherine T. Elliott

**Affiliations:** 1 Environmental Health Sciences Division, School of Public Health, University of California, Berkeley, Berkeley, California, USA; 2 Harvard Center for Population and Development Studies, Harvard T.H. Chan School of Public Health, Cambridge, Massachusetts, USA; 3 School of Population and Public Health, University of British Columbia, Vancouver, British Columbia, Canada; 4 Menzies Institute of Medical Research, University of Tasmania, Hobart, Tasmania, Australia; 5 Environmental Health Services, Department of Health and Human Services, Hobart, Tasmania, Australia; 6 Department of Environmental Health Sciences, Fielding School of Public Health, University of California, Los Angeles, Los Angeles, California, USA; 7 Department of Medicine, University of California, San Francisco, San Francisco, California, USA; 8 Office of the Chief Medical Officer of Health, Yukon Health and Social Services, Whitehorse, Yukon, Canada

## Abstract

**Background:**

Wildfire activity is predicted to increase in many parts of the world due to changes in temperature and precipitation patterns from global climate change. Wildfire smoke contains numerous hazardous air pollutants and many studies have documented population health effects from this exposure.

**Objectives:**

We aimed to assess the evidence of health effects from exposure to wildfire smoke and to identify susceptible populations.

**Methods:**

We reviewed the scientific literature for studies of wildfire smoke exposure on mortality and on respiratory, cardiovascular, mental, and perinatal health. Within those reviewed papers deemed to have minimal risk of bias, we assessed the coherence and consistency of findings.

**Discussion:**

Consistent evidence documents associations between wildfire smoke exposure and general respiratory health effects, specifically exacerbations of asthma and chronic obstructive pulmonary disease. Growing evidence suggests associations with increased risk of respiratory infections and all-cause mortality. Evidence for cardiovascular effects is mixed, but a few recent studies have reported associations for specific cardiovascular end points. Insufficient research exists to identify specific population subgroups that are more susceptible to wildfire smoke exposure.

**Conclusions:**

Consistent evidence from a large number of studies indicates that wildfire smoke exposure is associated with respiratory morbidity with growing evidence supporting an association with all-cause mortality. More research is needed to clarify which causes of mortality may be associated with wildfire smoke, whether cardiovascular outcomes are associated with wildfire smoke, and if certain populations are more susceptible.

**Citation:**

Reid CE, Brauer M, Johnston FH, Jerrett M, Balmes JR, Elliott CT. 2016. Critical review of health impacts of wildfire smoke exposure. Environ Health Perspect 124:1334–1343; http://dx.doi.org/10.1289/ehp.1409277

## Introduction

Wildfires are a global occurrence. Changes in temperature and precipitation patterns from climate change are increasing wildfire prevalence and severity (Westerling et al. 2006; Settele et al. 2014) resulting in longer fire seasons (Flannigan et al. 2013; Westerling et al. 2006) and larger geographic area burned (Gillett et al. 2004). Wildfire smoke contains many air pollutants of concern for public health, such as carbon monoxide (CO), nitrogen dioxide, ozone, particulate matter (PM), polycyclic aromatic hydrocarbons (PAHs), and volatile organic compounds (Naeher et al. 2007). Current estimated annual global premature mortality attributed to wildfire smoke is 339,000 (interquartile range of sensitivity analyses: 260,000–600,000) (Johnston et al. 2012), but the overall impact on public health in terms of respiratory, cardiovascular, and other morbidity effects is unknown. A better synthesis of current knowledge on the health effects of wildfire smoke is needed to guide public health responses.

Wildfire smoke epidemiology is an active area of research (Henderson and Johnston 2012) with new methods uncovering associations that were previously undetectable. Studies of health outcomes associated with wildfire smoke exposure tend to be retrospective and researchers have to rely on administrative health outcome data such as mortality or hospitalization records. Achieving adequate statistical power has been challenging because such severe outcomes are less common, fires tend to be episodic and short in duration, and exposed populations from individual events are often small. Many recent studies have increased statistical power by investigating very high exposure events that last for longer periods, large populations over many years in regions with frequent fires, more common health outcomes such as medication dispensations, or a combination of these methods.

Previous reviews of wildfire health impacts have either not included the full range of health end points associated with community exposure to wildfire smoke (Dennekamp and Abramson 2011; Henderson and Johnston 2012) or have summarized the literature without critical analysis of specific studies (Finlay et al. 2011; Liu et al. 2015; Youssouf et al. 2014). Our review follows a modified version of the systematic review methodology outlined in Woodruff and Sutton (2014) to analyze studies critically and to only evaluate the strongest evidence.

## Methods

**Figure 1:**
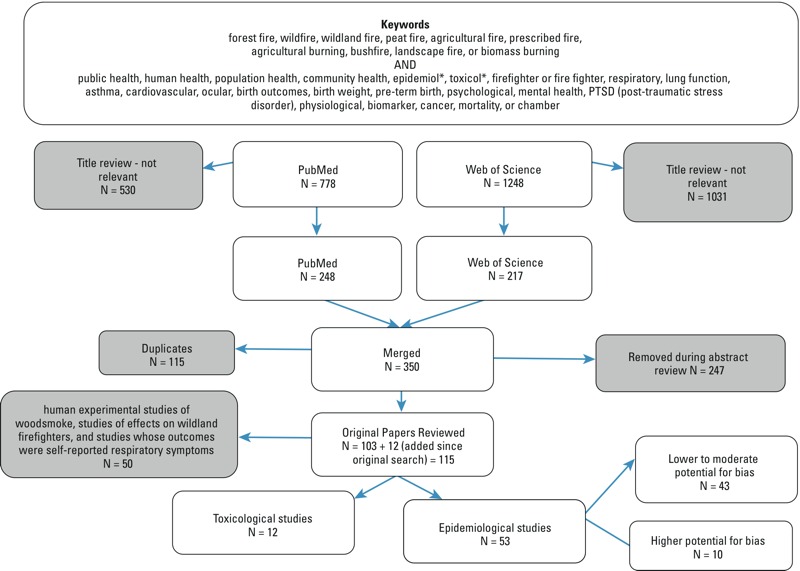
Review of studies flow chart.

We searched PubMed, Web of Science, and PsychInfo to identify scientific papers related to wildfire smoke exposure and relevant health outcomes. We conceptualized wildfires as those within the definition of landscape fires defined in Johnston et al. (2012). Our search strategy (Figure 1) yielded 778 journal articles in PubMed and 1,248 journal articles in Web of Science in November 2013. We then selected studies that potentially focused on human health effects related to wildfire smoke based on title and yielded 248 journal articles from PubMed and 217 from Web of Science. After discarding duplicates, 350 articles remained. PsychInfo did not yield any new peer-reviewed journal articles.

After reading abstracts, we removed articles if they assessed only exposure and not associated health effects, reported health surveillance outcomes without analysis of associations with exposure, did not analyze primary or secondary health data, did not adequately describe the exposure assessment or it was not clearly related to wildfire smoke, or were not published fully in English. This yielded 103 studies that we reviewed. We continually searched for new papers and subsequently added 12 more by August 2015. These papers included human experimental studies of woodsmoke, studies of effects on wildland firefighters, and studies whose outcomes were self-reported respiratory symptoms associated with wildfire smoke, but these are not included in this paper.

From the remaining epidemiological studies (*N* = 53), we extracted information and made an expert judgment on the risk of bias for each study based on their sample size, exposure assessment methods, control for potential confounding factors, and use of objective outcome measures (see Table S1). We deemed studies to have a lower risk of bias if there were no concerns in any of these categories, moderate risk if there were minor concerns in one or more categories, and higher risk if either there were multiple concerns about bias or if one concern was sufficiently large based on our collective judgment.

All evaluation of results from these studies is based on the authors’ interpretation of the reported findings in each paper. In this review “significant” means a 95% confidence interval (CI) that does not include the null, “suggestive” means a 95% CI that does include the null but would not with a slightly relaxed criterion such as a 90% CI, and “no association” means that the 95% CI includes the null with no indication of a relationship. We assumed that exposure to smoke from all types of landscape fires were comparable. We use the term wildfire to refer to all types of landscape fires.

Assessing human exposure to wildfire smoke is challenging for many reasons. Wildfires tend to occur in rural areas in which air pollution monitoring networks might be absent or less comprehensive than in cities. The studies we reviewed used various exposure assignment methods such as self-report, assignment to the nearest regulatory air pollution monitor, comparison of fire periods to non-fire periods, and use of satellite data or air quality modeling output. Heterogeneity of exposure assessment methods across studies (Table 1; see also Table S1) made a quantitative meta-analysis of effect estimates inappropriate. While publication bias could be present in this literature, we could not assess its extent due to the scarcity of studies for each health outcome.

**Table 1:** Findings from epidemiological research studies (*N* = 43) ordered by health outcome.

Outcome	Article	Exposure assessment type	Direction of association
Mortality
All	Sastry 2002	Monitored PM	↑↑
	Morgan et al. 2010	Monitored PM	↑↑
	Johnston et al. 2011	Smoky versus non-smoky days	↑↑
	Faustini et al. 2015	Smoky versus non-smoky days	↑↑
	Linares et al. 2015	Monitored PM	↑↑
	Shaposhnikov et al. 2014	Monitored PM	↑↑
Respiratory	Johnston et al. 2011	Smoky versus non-smoky days	↔
	Morgan et al. 2010	Monitored PM	↔
	Faustini et al. 2015	Smoky versus non-smoky days	↔
	Linares et al. 2015	Monitored PM	↔
Cardiovascular	Nunes et al. 2013	Modeled PM and satellite data	↑↑
	Faustini et al. 2015	Smoky versus non-smoky days	↑↑
	Johnston et al. 2011	Smoky versus non-smoky days	↑
	Morgan et al. 2010	Monitored PM	↔
	Linares et al. 2015	Monitored PM	↔
Respiratory morbidity
Lung function in people without asthma or bronchial hyperreactivity	Jacobson et al. 2012	Monitored PM	↓↓
	Jacobson et al. 2014	Monitored PM	↓↓
	Jalaludin et al. 2000	Monitored PM	↓↓
Physician visits	Lee et al. 2009	Monitored PM	↑↑
	Henderson et al. 2011	Monitored PM	↑↑
		Modeled PM	↑
		Binary satellite indicator of smoke	↑
	Moore et al. 2006	Temporal comparison	↑↑
	Mott et al. 2002	Temporal comparison	↑↑
	Lee et al. 2009	Monitored PM	↑↑
ED visits	Rappold et al. 2011	Temporal and spatial comparisons	↑↑
	Tham et al. 2009	Monitored PM	↑↑
	Thelen et al. 2013	Modeled PM	↑↑
	Johnston et al. 2014	Smoky versus non-smoky days	↑↑
Hospitalizations	Morgan et al. 2010	Monitored PM	↑↑
	Henderson et al. 2011	Monitored PM	↑↑
		Modeled PM	↑
		Binary satellite indicator of smoke	↑
	Johnston et al. 2007	Monitored PM	↑
	Delfino et al. 2009	PM monitoring, statistical modeling, and satellite information	↑↑
	Martin et al. 2013	Smoky versus non-smoky days	↑↑
	Chen et al. 2006	PM monitoring for categorical exposures	↑↑
	Cançado et al. 2006	PM monitoring	↑↑
	Mott et al. 2005	Temporal comparison	↑↑
	Ignotti et al. 2010	% annual hours > 80 μg/m^3^	↑↑
	Tham et al. 2009	Monitored PM	↔
Asthma
Lung function among people with asthma	Jacobson et al. 2012	Monitored PM	↔
	Jalaludin et al. 2000	Monitored PM	↔
	Vora et al. 2011	Temporal comparison	↔
	Wiwatanadate and Liwsrisakun 2011	Monitored PM	↔
Medications	Elliott et al. 2013	PM monitoring, statistical modeling, and satellite information	↑↑
	Yao et al. 2016	Modeled PM	↑↑
	Tse et al. 2015	Temporal and spatial comparisons	↑↑
	Vora et al. 2011	Temporal comparison	↑↑
	Johnston et al. 2006	Monitored PM	↑↑
	Arbex et al. 2000	Measurement of PM	↑
Physician visits	Henderson et al. 2011	Monitored PM	↑↑
		Modeled PM	↑↑
		Binary satellite indicator	↑
	Yao et al. 2014 2016	Monitored PM	↑↑
		Modeled PM	↑↑
ED visits	Johnston et al. 2002	Monitored PM	↑↑
	Rappold et al. 2011	Temporal and spatial comparisons	↑↑
	Duclos et al. 1990	Temporal comparison	↑↑
	Johnston et al. 2014	Smoky versus non-smoky days	↑↑
	Smith et al. 1996	Temporal comparison	↑
	Tse et al. 2015	Temporal and spatial comparisons	↔
Hospitalizations	Morgan et al. 2010	Monitored PM	↑↑
	Delfino et al. 2009	PM monitoring, statistical modeling, and satellite information	↑↑
	Arbex et al. 2007	PM monitoring	↑↑
	Martin et al. 2013	Smoky versus non-smoky days	↑↑
	Johnston et al. 2007	Monitored PM	↑
	Tse et al. 2015	Temporal and spatial comparisons	↔
COPD
Physician visits	Yao et al. 2016	Monitored PM	↑↑
		Modeled PM	↑↑
ED visits	Rappold et al. 2011	Temporal and spatial comparisons	↑↑
	Duclos et al. 1990	Temporal comparison	↑↑
	Johnston et al. 2014	Smoky versus non-smoky days	↑↑
Hospitalizations	Morgan et al. 2010	Monitored PM	↑↑
	Johnston et al. 2007	Monitored PM	↑↑
	Delfino et al. 2009	PM monitoring, statistical modeling, and satellite information	↑↑
	Martin et al. 2013	Smoky versus non-smoky days	↑↑
	Mott et al. 2005	Temporal comparison^*a*^	↑↑
Respiratory infections
Physician visits	Yao et al. 2016	Monitored PM^*b*^	↑↑
		Modeled PM^*b*^	↔
		Monitored PM^*c*^	↑↑
		Modeled PM^*c*^	↑↑
	Henderson et al. 2011	Monitored PM^*d*^	↔
ED visits	Duclos et al. 1990	Temporal comparison^*b*^	↑↑
	Rappold et al. 2011	Temporal and spatial comparisons^*b*^	↑
Hospitalizations	Johnston et al. 2007	Monitored PM	↔
Pneumonia and bronchitis
ED visits	Rappold et al. 2011	Temporal and spatial comparisons	↑↑
	Johnston et al. 2014	Smoky versus non-smoky days	↔
Hospitalizations	Delfino et al. 2009	PM monitoring, statistical modeling, and satellite information	↑↑
	Morgan et al. 2010	Monitored PM	↑↑
	Martin et al. 2013	Smoky versus non-smoky days	↑
	Duclos et al. 1990	Temporal comparison^*e*^	↑↑
Cardiovascular morbidity
Physician visits	Henderson et al. 2011	Monitored PM	↔
		Modeled PM	↔
		Binary satellite indicator	↔
	Moore et al. 2006	Temporal comparison	↔
	Lee et al. 2009	Monitored PM	↔
	Yao et al. 2016	Monitored PM	↓↓
		Modeled PM	↔
ED visits	Rappold et al. 2011	Temporal and spatial comparisons	↔
	Johnston et al. 2014	Smoky versus non-smoky days	↔
Hospitalizations	Morgan et al. 2010	Monitored PM	↔
	Hanigan et al. 2008	PM estimated from visibility data	↔
	Henderson et al. 2011	Monitored PM	↔
		Modeled PM	↔
		Binary satellite indicator	↔
	Johnston et al. 2007	Monitored PM	↔
	Martin et al. 2013	Smoky versus non-smoky days	↔
CHF
ED visits	Rappold et al. 2011	Temporal and spatial comparisons	↑↑
Hospitalizations	Delfino et al. 2009	PM monitoring, statistical modeling, and satellite information	↑
	Morgan et al. 2010	Monitored PM	↔
	Martin et al. 2013	Smoky versus non-smoky days	↔
Cardiac arrest
Out-of-hospital	Dennekamp et al. 2015	PM monitoring	↑↑
	Haikerwal et al. 2015	Modeled PM	↑↑
ED visits	Johnston et al. 2014	Smoky versus non-smoky days	↔
Acute MI
ED visits	Haikerwal et al. 2015	Modeled PM	↔
Hospitalizations	Haikerwal et al. 2015	Modeled PM	↑↑
IHD
Physician visits	Lee et al. 2009	Monitored PM	↑↑
ED visits	Johnston et al. 2014	Smoky versus non-smoky days	↑
	Haikerwal et al. 2015	Modeled PM	↑
Hospitalizations	Mott et al. 2005	Temporal comparison	↑
	Haikerwal et al. 2015	Modeled PM	↑
	Morgan et al. 2010	Monitored PM	↔
	Delfino et al. 2009	PM monitoring, statistical modeling, and satellite information	↔
	Johnston et al. 2007	Monitored PM	↓↓ and ↑↑^*f*^
	Martin et al. 2013	Smoky versus non-smoky days	↔
Hypertension
Physician visits	Henderson et al. 2011	Monitored PM	↔
Hospitalizations	Arbex et al. 2010	PM monitoring	↑↑
Cardiac dysrhythmias/arrhythmias			
ED visits	Johnston et al. 2014	Smoky versus non-smoky days	↔
Hospitalizations	Delfino et al. 2009	PM monitoring, statistical modeling, and satellite information	↔
	Martin et al. 2013	Smoky versus non-smoky days	↔
Cerebrovascular disease
ED visits	Johnston et al. 2014	Smoky versus non-smoky days	↔
Hospitalizations	Delfino et al. 2009	PM monitoring, statistical modeling, and satellite information	↑
	Morgan et al. 2010	Monitored PM	↔
Angina
Dispensations of fast-acting nitroglycerin	Yao et al. 2016	Monitored PM	↑↑
ED visits	Haikerwal et al. 2015	Modeled PM	↑
Hospitalizations	Haikerwal et al. 2015	Modeled PM	↔
Birth outcomes
Birth weight	Holstius et al. 2012	Temporal comparison	↓↓
Proportion of cohort surviving	Jayachandran 2009	Satellite data	↓↓
Low birth weight	Cândido da Silva et al. 2014	Monitored PM	↑↑
Mental health
Physician visits	Moore et al. 2006	Temporal comparison	↔
Hospitalizations	Duclos et al. 1990	Temporal comparison	↔
^***a***^Asthma and COPD combined. ^***b***^Upper respiratory infections. ^***c***^Lower respiratory infections. ^***d***^Upper respiratory infections and acute bronchitis combined. ^***e***^Bronchitis alone. ^***f***^Significantly elevated for indigenous population, but significantly lower risk for whole population. ↔ No association. ↑ Suggestive increase. ↑↑ Significant increase. ↓↓ Significant decrease.			

## Results

Our review covers the following health outcomes: mortality, respiratory morbidity, cardiovascular morbidity, birth outcomes, and mental health. We further discuss the evidence from toxicological studies and for susceptible population subgroups. Table S1 provides more details on reviewed studies.

After review of 53 epidemiological papers, we evaluated 27 as having lower potential for bias, 17 as moderate potential for bias and 10 as higher potential for bias. Of the 10 deemed to have higher risk of bias, 4 did not adequately adjust for important covariates (Azevedo et al. 2011; Cooper et al. 1994; Prass et al. 2012; Resnick et al. 2015), 2 were likely underpowered due to small sample size (Cooper et al. 1994; Vedal and Dutton 2006), 3 used retrospective self-report for exposure assessment with high potential for bias (Ho et al. 2014; McDermott et al. 2005; Marshall et al. 2007), and the exposure assessment in 2 other studies was not clearly related to smoke from wildfires (Analitis et al. 2012, Caamano-Isorna et al. 2011). The remaining 43 studies deemed to have low to moderate risk of bias are discussed below. More detail on the findings from each study is provided in Table S2.

### Mortality

Growing evidence from the more recent, adequately statistically powered studies demonstrates associations between wildfire smoke exposure and all-cause mortality, but more studies are needed to determine whether specific causes of mortality are most affected.

A study of the 1997 southeast Asian wildfire found an increase in mortality in Malaysia associated with a measure of visibility and measured PM_10_ (PM ≤ 10 μm in aerodynamic diameter) both linearly and with various discrete levels of PM_10_ (Sastry 2002). A study of the 2010 heat wave and wildfires in Moscow reported findings of an interaction between high temperatures and high PM_10_ on deaths and that smoke exposure was responsible for about 29% of the 10,859 excess deaths during the 44-day heat wave (Shaposhnikov et al. 2014). A cross-sectional analysis of cardiovascular mortality among people older than 65 years in the Brazilian Amazon, where the predominant source of air pollution is from wildfires, found a significant association between the percentage of hours of PM_2.5_ over 25 μg/m^3^ and cardiovascular mortality (Nunes et al. 2013).

The most recent studies of wildfire smoke and mortality take advantage of long time series data and provide growing evidence of significant increases in mortality. A study of 13.5 years of data including 48 days affected by wildfire smoke in Sydney, Australia, demonstrated a significant increase in mortality associated with smoke-affected days (Johnston et al. 2011). An earlier study of mortality in Sydney, using 8 years of data, found a suggestive increase in mortality associated with wildfire-related PM_10_ (Morgan et al. 2010). A meta-analysis of data from 2003 to 2010 in 10 cities in southern Europe found increases in cardiovascular mortality associated with PM_10_ that were stronger on smoke-affected days than on non-affected days, but smoke was not significantly associated with respiratory mortality (Faustini et al. 2015). In Madrid, mortality, but not specifically respiratory or cardiovascular mortality, was associated with PM_10_ on days with advection events associated with biomass burning (Linares et al. 2015). Further multi-year studies in regions regularly affected by wildfire smoke could help clarify if specific causes of mortality are associated with wildfire smoke exposure.

### Respiratory Morbidity

Epidemiological studies have demonstrated significant associations between wildfire smoke exposure and declines in lung function among non-asthmatic children (Jacobson et al. 2012, 2014), and increases in physician visits for respiratory problems (Henderson et al. 2011; Lee et al. 2009; Moore et al. 2006; Mott et al. 2002), respiratory emergency department (ED) visits (Johnston et al. 2014; Rappold et al. 2011; Tham et al. 2009; Thelen et al. 2013) and respiratory hospitalizations (Cançado et al. 2006; Chen et al. 2006; Delfino et al. 2009; Henderson et al. 2011; Ignotti et al. 2010; Martin et al. 2013; Morgan et al. 2010; Mott et al. 2005). Findings for specific respiratory end points are reviewed below.

***Asthma.*** Evidence from multiple epidemiological studies demonstrates that wildfire smoke exposure contributes to exacerbations of asthma. Studies have documented increased physician visits (Henderson et al. 2011; Yao et al. 2016), ED visits (Duclos et al. 1990; Johnston et al. 2002, 2014; Rappold et al. 2011) and hospitalizations (Arbex et al. 2007; Delfino et al. 2009; Martin et al. 2013; Morgan et al. 2010; Mott et al. 2005) for asthma associated with wildfire smoke exposure. Some studies found suggestive increases in asthma ED visits (Smith et al. 1996) and asthma hospital admissions (Johnston et al. 2007); these studies may have lacked statistical power due to short time periods (Smith et al. 1996) or small affected populations (Johnston et al. 2007). Another study did not find a significant increase in ED visits or hospitalizations among a cohort of asthmatic children in the year after large wildfires in San Diego, California, compared to the year prior to those fires (Tse et al. 2015).

Four studies demonstrated no significant acute changes in lung function among people with asthma related to PM from wildfires (Jacobson et al. 2012; Jalaludin et al. 2000; Vora et al. 2011; Wiwatanadate and Liwsrisakun 2011), although significant declines in lung function were found among those without asthma (Jacobson et al. 2012) and children without bronchial hyper-reactivity (Jalaludin et al. 2000). One possible explanation for these counter-intuitive findings is increased use of rescue medication in response to elevated levels of smoke among those diagnosed with asthma as was found in one (Vora et al. 2011) of two studies (Vora et al. 2011; Jacobson et al. 2012) that investigated this mechanism.

Other studies documented associations between medication usage for obstructive lung disease and wildfire smoke exposure. Both usage of reliever medication and initiation of oral steroid use were associated with wildfire smoke in a panel study of adults and children in Australia (Johnston et al. 2006). People with asthma reported elevated levels of rescue medication usage during a wildfire in Southern California (Vora et al. 2011). Dispensations of reliever medications were related to metrics of wildfire smoke exposure in British Columbia (Elliott et al. 2013; Yao et al. 2016). Researchers found increases in physician-dispensed short-acting beta-agonists but not physician-prescribed oral corticosteroids for children with asthma in years after two catastrophic wildfires in southern California compared to the year prior to each wildfire (Tse et al. 2015). An association between visits to hospitals for inhalation therapy and daily mass of air particle sediment collected in four nearby water containers was found during one sugarcane-burning season in Brazil (Arbex et al. 2000).

All previously mentioned studies examined exacerbations of asthma, whereas only one study investigated incident asthma related to wildfire smoke. Methodological concerns in that portion of the study suggest a high potential for bias as new diagnoses occurring after, but not during, two large wildfire episodes were included (Tse et al. 2015).

***Chronic obstructive pulmonary disease (COPD).*** Epidemiological evidence of associations between wildfire smoke exposure and exacerbation of COPD is mounting. Elevated rates of hospitalizations (Delfino et al. 2009; Johnston et al. 2007; Martin et al. 2013; Morgan et al. 2010; Mott et al. 2005), ED visits (Duclos et al. 1990; Johnston et al. 2014; Rappold et al. 2011), and physician visits for COPD (Yao et al. 2016) have been associated with wildfire smoke exposure. Additionally, the findings of increased reliever medication dispensing during wildfire smoke exposure in British Columbia may indicate increases in COPD or asthma exacerbations (Elliott et al. 2013; Yao et al. 2016).

***Respiratory infections.*** The evidence for associations between wildfire smoke exposure and respiratory infections is inconsistent. Duclos et al. (1990) found a higher rate of ED visits for respiratory infections during major wildfires in California compared to a reference period. Rappold et al. (2011) found a suggestive increase in ED visits for upper respiratory infections in smoke-affected counties in North Carolina during peat fires compared to a reference period and this temporal increase was not found in non-smoke-affected counties. Henderson et al. (2011) and Yao et al. (2016), however, found no association between wildfire smoke exposure and physician visits for upper respiratory infections in British Columbia. Johnston et al. (2007) reported no association between PM predominantly from wildfires and hospitalizations for respiratory infections in Australia.

The evidence does suggest an association between wildfire smoke and acute bronchitis and pneumonia, however. Although Johnston et al. (2014) did not find an association between ED visits for pneumonia and bronchitis associated with wildfire smoke in Australia, most other studies did. Yao et al. (2016) found significant increases in physician visits for lower respiratory infections associated with PM_2.5_ over 10 fire seasons in British Columbia. Rappold et al. (2011) documented increased ED visits for pneumonia and acute bronchitis associated with exposure to smoke from a peat fire. Duclos et al. (1990) found higher rates of hospitalization for bronchitis during a wildfire compared to a reference period. Moreover, Martin et al. (2013) reported associations between days with high levels of bushfire smoke and hospitalizations for pneumonia and acute bronchitis in Newcastle, Australia, although this association was not found in the larger city of Sydney; the authors attribute this to lack of precision in estimates of specific respiratory outcomes. Two studies have documented similar associations between wildfire smoke and background PM with bronchitis and pneumonia (Delfino et al. 2009; Morgan et al. 2010), suggesting that effects of wildfire and urban PM on these outcomes are similar.

### Cardiovascular Morbidity

Results from studies of associations between cardiovascular outcomes and wildfire smoke exposure are inconsistent. Many studies of wildfire smoke exposure have found no associations with grouped cardiovascular disease outcomes (Hanigan et al. 2008; Henderson et al. 2011; Johnston et al. 2007, 2014; Lee et al. 2009; Martin et al. 2013; Moore et al. 2006; Morgan et al. 2010; Rappold et al. 2011; Yao et al. 2016), although a few have documented evidence for specific end points. Rates of out-of-hospital cardiac arrests were associated with wildfire-related PM_2.5_ in Australia (Dennekamp et al. 2015; Haikerwal et al. 2015). Hospitalizations but not ED visits for acute myocardial infarctions (MI) were associated with wildfire-related PM_2.5_ during the same fires (Haikerwal et al. 2015). ED visits for congestive heart failure (CHF) were associated with wildfire smoke exposure from a peat fire in North Carolina (Rappold et al. 2011), but only a suggestive association was found for CHF hospitalizations and PM_2.5_ during a wildfire in southern California (Delfino et al. 2009). Johnston et al. (2014) did not find any association between wildfire smoke and ED cardiac failure. Other studies have found no associations between wildfire smoke exposure and CHF (Martin et al. 2013; Morgan et al. 2010) or cardiac dysrhythmias (Delfino et al. 2009; Johnston et al. 2014; Martin et al. 2013). And no associations were found in the one study that investigated angina in relation to wildfire PM_2.5_ (Haikerwal et al. 2015).

Study results are also mixed for ischemic heart disease (IHD). Higher counts of hospitalizations for IHD than expected based on historical data were found in Sarawak, Malaysia, during the prolonged very high PM levels of the 1997 Southeast Asian wildfires (Mott et al. 2005). ED visits for IHD were higher on smoke-affected days in Sydney, Australia (Johnston et al. 2014), but two other studies in Australia (Martin et al. 2013; Morgan et al. 2010) and one in California (Delfino et al. 2009) reported no associations for IHD hospital admissions. A study in Darwin, Australia, found increased risk of IHD hospitalizations only among the indigenous population, whereas the results suggested an inverse association among the whole population (Johnston et al. 2007). Researchers also found a positive association between PM_10_ during a wildfire and clinic visits for IHD in a Native American reservation in California (Lee et al. 2009).

Very few studies have investigated other cardiovascular outcomes, making definitive conclusions difficult. Arbex et al. (2010) found increases in hospitalizations for hypertension associated with exposure to total suspended particles over 2 years within a community seasonally exposed to smoke from burning sugarcane, but there was no clear difference in this finding between burning and non-burning periods, which implies that the relationship may not be due to the source of the particles. Henderson et al. (2011) did not find any relationship between PM_10_ during a wildfire and physician visits for hypertension. One (Delfino et al. 2009) of three (Delfino et al. 2009; Morgan et al. 2010; Johnston et al. 2014) studies to investigate cerebrovascular disease or stroke found a suggestive association with wildfire smoke exposure.

Too few studies and too many inconsistencies in findings exist to determine whether wildfire smoke exposure is associated with specific cardiovascular outcomes, despite evidence that exposure to ambient PM is associated with increased risk of cardiovascular morbidity (Brook et al. 2010).

### Birth Outcomes

Corroborative evidence suggests that wildfire smoke exposure effects on birth outcomes are plausible. For example, a growing literature exists on associations between adverse birth outcomes and exposure to ambient air pollution (Woodruff et al. 2010), to wood smoke from household cooking and heating in developing countries (e.g., Lakshmi et al. 2013) and to household heating in developed countries (Gehring et al. 2014). While these exposures are chronic compared to the more acute nature of exposure to smoke from some wildfires, some studies have demonstrated links between wildfire smoke exposure and birth outcomes. Holstius et al. (2012) found lower birth weights, overall and for the second and third trimesters specifically, for babies that gestated during the 2003 southern California wildfires compared to babies from the same region born before or more than 9 months after the fires. Jayachandran (2009) found that prenatal smoke exposure from the 1997 Southeast Asian wildfire in the third trimester was the most important predictor of ‘missing’ children from the Indonesian 2000 Census, the only way to estimate early life deaths from the scant data in Indonesia. Pregnant women exposed to very high levels of PM_2.5_ from agricultural burning in the Brazilian Amazon had higher rates of low birthweight babies compared to those exposed to lower levels (Cândido da Silva et al. 2014).

### Mental Health Outcomes

Although many studies have documented evidence of psychological impairment related to wildfires (e.g. Papanikolaou et al. 2011), few have investigated smoke exposure as a cause. We found six studies that investigated the association between objective mental health impacts and wildfire smoke exposure; however, four of those were deemed to have higher potential for bias (Ho et al. 2014; McDermott et al. 2005; Marshall et al. 2007; Caamano-Isorna et al. 2011). In the two studies that remain, one found no increase in physician visits for mental illness associated with PM during the 2003 wildfire season in British Columbia (Moore et al. 2006) and the other found no increase in mental health hospitalizations during the 1987 California fires compared to a reference period (Duclos et al. 1990).

### Toxicological Studies

A major pathway by which PM causes respiratory effects is through pulmonary oxidative stress and inflammation (Nakayama Wong et al. 2011). Systemic responses are the main pathways through which PM is thought to influence cardiovascular health. These are hypothesized to be induced either directly by the movement of pro-inflammatory, pro-coagulation, and pro-oxidant components of PM to the circulation, indirectly as a consequence of the pulmonary changes induced by PM, or through PM-mediated changes in the autonomic nervous system (Brook et al. 2010; Delfino et al. 2010).

*In vivo* animal studies of wildfire-derived PM exposure compared to controls have demonstrated increased oxidative stress and cell death in mice (Williams et al. 2013), and lower counts of lung macrophages, higher levels of inflammatory cells and cytokines, and greater antioxidant depletion in a study of smoke from a California wildfire in a mouse model (Wegesser et al. 2009, 2010).Similarly, increased respiratory inflammation and reduced lung mechanics compared with controls was documented from a mouse study of biomass smoke from burning sugarcane in Brazil (Mazzoli-Rocha et al. 2008). *In vivo* studies in humans have also demonstrated increased inflammatory responses, specifically elevated band neutrophil counts in peripheral blood (Tan et al. 2000) and elevated cytokines (van Eeden et al. 2001) associated with air pollution levels during the 1997 Southeast Asian wildfires.

*In vitro* studies have documented increased inflammation in rat alveolar macrophages exposed to PM_2.5_ from prescribed fires (Myatt et al. 2011) and in human bronchial epithelial cells exposed to wildfire-derived PM_2.5_ compared to cells exposed to ambient PM (Nakayama Wong et al. 2011). After exposure to wildfire-derived PM, human lung epithelial cells showed declines in glutathione, an important antioxidant (Pavagadhi et al. 2013); mouse peritoneal monocytes showed increased hydrogen peroxide production and oxygen radical generation (Leonard et al. 2007); and mouse macrophages (Franzi et al. 2011), rat macrophages (Myatt et al. 2011), and human lung epithelial cells (Pavagadhi et al. 2013) had increased cell death.

Oxidative stress can also lead to DNA damage. All size fractions of PM extracted from wildfire smoke caused DNA damage in mouse peritoneal monocytes (Leonard et al. 2007). Studies in regions near sugarcane burning in the Brazilian Amazon observed higher numbers of micronucleated cells, a measure of genotoxicity, in buccal cells from children in highly smoke-affected areas compared to children in a control community (Sisenando et al. 2012); however, it is unclear if the higher pollution in the study communities was solely due to agricultural burning because two factories are located in the exposed but not in the control region. Another study found more micronucleated buccal cells in sugarcane workers compared to nearby hospital administrative workers (Silveira et al. 2013), but the authors do not mention any control for other differences in these two populations that could explain this finding.

A recent study demonstrated the potential for early life exposure to wildfire smoke to confer immune effects, measured as reduced cytokine synthesis in peripheral blood cells, lasting into adolescence in Rhesus macaque monkeys (Miller et al. 2013). Short-term inhalation of wood smoke in general and not specifically from a wildfire can compromise lung immune responses, which may be one reason for the observed increased likelihood of lung infections in children exposed to wood smoke (Zelikoff et al. 2002). There is therefore growing evidence to support the theory that incidence of respiratory infections can be increased by exposure to wildfire smoke.

In summary, existing toxicological evidence supports potential respiratory and cardiovascular health effects of wildfire smoke exposure. The body of evidence, however, is relatively small compared to toxicological studies of general PM.

### Vulnerable Populations

Few epidemiological studies have investigated whether specific populations are more susceptible to wildfire smoke exposure than the general population. Susceptibility factors investigated include those related to lifestage, pre-existing disease, socioeconomic status (SES), and ethnicity. Unless otherwise stated, all subgroup differences are based on observed changes in the magnitudes of point estimates, not on significance tests.

The findings for differential effects by age are inconclusive. A study of PM_10_ exposure in Malaysia from the 1997 Southeast Asian wildfires found higher rates of mortality among people 65–74 years old compared to others; a smaller suggestive effect was found among those ≥ 75 years old (Sastry 2002). People ≥ 65 years old had higher rates of respiratory hospitalizations compared to younger adults exposed to biomass burning in the Brazilian Amazon (Ignotti et al. 2010) and wildfire smoke in Australia (Morgan et al. 2010). Such older adults were also found to have higher rates of hospitalization for asthma than their younger counterparts during California wildfires (Delfino et al. 2009), and higher rates of out-of-hospital cardiac arrests and hospitalizations for IHD in Victoria, Australia (Haikerwal et al. 2015).

Other studies, however, have found higher effects for younger adults than for older adults. Wildfire PM-related respiratory admissions during Indonesian wildfires exceeded predictions for 40- to 64-year-olds but not for those ≥ 65 years (Mott et al. 2005). Similarly, ED visits for COPD, and pneumonia and acute bronchitis were more strongly associated with peat fire smoke among people < 65 years old compared to people ≥ 65 in North Carolina (Rappold et al. 2011). Although respiratory physician visits were associated with PM_10_ among people 60–70 years old and among those ≥ 80 in a British Columbia wildfire, younger adults exhibited stronger associations (Henderson et al. 2011). No differences were found in either of the two studies that investigated differential effects by age for cardiovascular outcomes (Morgan et al. 2010, Henderson et al. 2011).

Children with asthma did not experience increased respiratory symptoms or medication use during Australian wildfires, whereas adults did (Johnston et al. 2006). Similarly, the highest PM-related increase in physician visits for asthma during a wildfire in British Columbia was found for adults (Henderson et al. 2011), as was true for ED visits for asthma on smoke-affected days in Australia (Johnston et al. 2014). Asthma hospitalizations among children ages 0–5 years were more strongly associated with wildfire PM_2.5_ exposure than were asthma hospitalizations for both older children and adults < 65 years old during a California wildfire; but the greatest association was found for people ≥ 65 years (Delfino et al. 2009).

Some studies have used previous health care utilization as a measure of pre-existing health conditions. One study found no effect modification by number of physician visits in the previous year (Henderson et al. 2011). In contrast, people ≥ 65 years old who were hospitalized for any cardiorespiratory outcome in the first half of the year were at increased risk of being hospitalized during the 1997 Southeast Asian fires compared with similar temporal comparisons in previous years without fires (Mott et al. 2005). Pre-existing cardiac or respiratory conditions may plausibly increase vulnerability to wildfire smoke exposure; however, the available evidence is currently inconclusive.

A recent study found that body mass index modified the association of wildfire smoke exposure on exacerbations of asthma, as measured by prevalence of physician-dispensed short-acting beta-agonists for children with asthma in southern California (Tse et al. 2015).

Few studies have investigated how socio-economic status (SES) influences responses to wildfire smoke exposure. Henderson et al. (2011) noted findings of no effect modification by neighborhood SES on associations between wildfire smoke exposure and physician visits in British Columbia, Canada, but detailed results were not presented. In contrast, during a North Carolina peat fire, North Carolina counties with lower SES had higher rates of ED visits for asthma and CHF compared to counties with higher SES (Rappold et al. 2012). Similarly, in Indonesia, districts with lower food consumption demonstrated larger adverse associations between smoke exposure and survival of birth cohorts than those with higher household food consumption (Jayachandran 2009).

To our knowledge only one ethnic subgroup has been studied in relation to differential health outcomes associated with wildfire smoke exposure. Indigenous people in Australia experienced higher rates of hospitalization for respiratory infections (Hanigan et al. 2008), and IHD (Johnston et al. 2007) associated with exposure to bushfire smoke than non-indigenous people. This effect may be explained by underlying health status, access to medical services, or other social characteristics in this group (Martin et al. 2013).

## Discussion

Our critical review demonstrated consistent evidence of associations between wildfire smoke exposure with general respiratory morbidity and with exacerbations of asthma and COPD (Table 1). Mounting epidemiological evidence and plausible toxicological mechanisms suggest an association between wildfire smoke exposure and respiratory infections, but inconsistencies remain. Increasing evidence suggests an association between wildfire smoke exposure and all-cause mortality, especially from more recent, higher-powered studies (e.g., Johnston et al. 2011; Morgan et al. 2010; Faustini et al. 2015). The current evidence for cardiovascular morbidity from wildfire smoke exposure remains mixed; many studies are inconclusive or negative, but some have demonstrated significant increases for specific cardiovascular outcomes, such as cardiac arrests. Toxicological findings are consistent with cardiac effects through evidence of systemic inflammation and increased coagulability. Most of the other end points of interest, including birth outcomes, mental health, and cancer have not been sufficiently studied.

Our review highlights the lack of information about which populations are most susceptible to wildfire smoke exposure. People already diagnosed with asthma or COPD are more susceptible. We found inconsistent evidence of differential effects by age or SES. Two studies have suggested differential effects by Australian indigenous status with no investigation of other ethnic groups.

Many gaps exist in understanding the public health implications of exposure to wildfire smoke. Larger studies with greater statistical power and more spatially refined exposure assessments are needed to better characterize impacts on mortality, cardiovascular disease, birth outcomes, and mental health effects. Currently, evidence exists of exacerbation, but not incidence, of asthma and COPD from wildfire smoke exposure. In temperate parts of the world, where wildfire smoke exposure is episodic, it is unlikely that changes in asthma incidence would be observed. Studies have not been conducted in populations more chronically exposed to wildfire smoke. Additionally, other health outcomes associated with wildfire smoke exposure have not yet been sufficiently studied, such as otitis media, which has been associated with exposure to secondhand tobacco smoke (Kong and Coates 2009), air pollution from woodsmoke (MacIntyre et al. 2011) and recently wildfire smoke (Yao et al. 2016). Human experimental studies of exposures to wildfire smoke could help clarify biological mechanisms. Very little information exists on health effects associated with measures of pollutants in wildfire smoke other than PM, such as ozone or PAHs. Although this review combined results from studies of various types of fires, it is possible that smoke originating from peat fires, forest fires, grassland fires, and agricultural burning could lead to differential health effects due to different constituents in the smoke. To our knowledge, no studies have yet investigated chronic exposure to wildfire smoke, but many populations in Southeast Asia, Africa, and Latin America are exposed regularly for extended periods (Johnston et al. 2012).

Characterization of the exposure–response function is critical for setting smoke levels for public health warnings or interventions, and it is not yet known whether current levels based on undifferentiated PM sufficiently characterize the effects of wildfire smoke. Four studies (Arbex et al. 2010; Chen et al. 2006; Johnston et al. 2002; Sastry 2002) have attempted to identify effects at different exposure levels, but these studies are hard to compare because of differences in exposure assessment methods, health outcomes, types of fires, and population susceptibilities.

## Conclusions

We found consistent evidence of associations between wildfire smoke exposure and respiratory morbidity in general, and specifically for exacerbations of asthma and COPD. Growing evidence suggests associations with respiratory infections and all-cause mortality. More research is needed to determine whether wildfire smoke exposure is consistently associated with cardiovascular effects, specific causes of mortality, birth outcomes, and mental health outcomes. Research into which populations are most susceptible to health effects from wildfire smoke exposure is also needed to inform public health planning for future wildfires.

## Supplemental Material

(916 KB) PDF
