# Supplementary material for: Critical Review of Health Impacts of Wildfire Smoke Exposure
Source: Environ Health Perspect. 2016 Apr 15;124(9):1334–43. doi: 10.1289/ehp.1409277 (PMC5010409; doi:10.1289/ehp.1409277)
Supplement: (916 KB) PDF [file ehp.1409277.s001.acco.pdf]

Environ Health Perspect
DOI: 10.1289/ehp.1409277

Note to readers with disabilities: *EHP* strives to ensure that all journal content is accessible to all readers. However, some figures and Supplemental Material published in *EHP* articles may not conform to 508 standards due to the complexity of the information being presented. If you need assistance accessing journal content, please contact ehp508@niehs.nih.gov. Our staff will work with you to assess and meet your accessibility needs within 3 working days.

Supplemental Material

Critical Review of Health Impacts of Wildfire Smoke Exposure

Colleen E. Reid, Michael Brauer, Fay Johnston, Michael Jerrett, John R. Balmes, and Catherine T. Elliott

Table of Contents

Table S1. Assessment of Risk of Bias for all epidemiological studies reviewed (N=53)
Table S2. Effect estimates for original epidemiological research studies (N=53), regardless of level of potential bias, ordered by health outcome and type of effect estimate.
References

Table S1: Assessment of Risk of Bias for all epidemiological studies reviewed (N=53)

Article	Fire Event/Location	Sample Size	Exposure Assessment Method	Exposure Levels	Covariates controlled for	Outcomes	Risk of Bias	Comment on Risk of Bias
Analitis et al. 2011	Athens 1998-2004	1071 days; Average number of deaths per day = 73 and	Categorized days with large fires (fires greater than 30,000,000 m^3), medium fires (1,000,001-30,000,000 m^3), and small fires (10,000 – 1,000,000 m^3), compared to days with no fires	No smoke levels reported	PM, temperature, heat wave day, RH, wind speed, wind direction, day of week, holidays and seasonal and long-term trend.	mortality, all-cause	higher	Exposure assessment method may not be related to smoke exposure; adjustment for black smoke may have attenuated impacts of wildfire-generated smoke
Arbex et al. 2000	Araraquara, Brazil, sugarcane burning season June 1-August 31, 1995	97 days with an average number of hospital visits for inhalation therapy of 22	Gravimetric analysis of particles centrifuged daily from water in receptacles placed at two sites in Araraquara.	12.9 ±7.0 mg sediment per day	Seasonality, temperature, day of week, precipitation	Hospital visits for inhalation therapy	moderate	Exposure assessment method is unique to this study and only yielded the largest particles, as noted by the authors
Arbex et al. 2007	Araraquara, Saõ Paulo State, Brazil, from 23 March 2003 to 27 July 2004	493 days and a total of 640 asthma hospitalizations	TSP from one monitor downtown	Mean TSP = 46.8 ± 26.4 $\mu g/m^3$	long-term trend, temperature, humidity	hospitalizations, asthma	lower	
Arbex et al. 2010	Araraquara, Brazil 23 March 2003 to 27 July 2004	493 days and mean of 2.5 hypertension – related hospital admissions per day	TSP from one monitor downtown	Burning period TSP mean 56.866 ± 25.07 $\mu g/m^3$	long-term trend, temp, RH	hospitalizations, hypertension	lower	
Azevedo et al. 2011	Portugal 2005	350 days	One central monitor	42 days in 2005 had ozone levels over 180 $\mu g/m^3$	Ozone, PM_{10}, SO_2, NO, CO, NO_2, $PM_{2.5}$	Respiratory and cardiovascular hospitalizations	higher	Models not adjusted for temporal trend, seasonality, day of week, or temperature effects. Multipollutant

								models without dealing with collinearity.
Caamano-Isorna et al. 2011	August 2006 Galician Fires	4212 municipality-months (156 municipalities *27 months); did not give average daily doses of each drug per 1000 inhabitants for these municipalities but did for all of Spain: 46.51 for anxiolytics, 22.19 for hypnotics, and 45 for drugs for obstructive airway disease	Number of wildfires within a municipality used to classified municipalities into no exposure (0-3 wildfires), medium exposure (4-10) and high exposure (more than 10).	No air quality exposure assessment	Interaction of exposure and time period, time trend, sex and age by stratification	drug dispensations for anxiolytics and for obstructive airway diseases	higher	Exposure assessment of number of fires in a region may not represent fire smoke exposure and no assessment of air quality
Cançado et al. 2006	Piracicaba in southeast Brazil. From April 1997 through March 1998	306 days; mean daily hospital admissions for children was 2.2 and for elderly was 0.9	PM_{10}, $PM_{2.5}$ and speciated PM information that was used in factor analysis to determine sources	Not reported for the biomass burning factor	long-term trend, day of week, temperature, RH	hospitalization, respiratory	lower	
Candido da Silva et al. 2014	Retrospective cohort of births in cities in Mato Grosso State, Brazil from July 1, 2004 and December 31, 2005	6147 full-term live births	$PM_{2.5}$ from one monitoring station	Average $PM_{2.5}$ levels in 2004 of 21.7 ± 35.2 μg/m^3 and in 2005 of 18.1 ± 33.7 μg/m^3	Sex, mother's education, prenatal visits, type of delivery, and age group	Low birth weight	lower	
Chen et al. 2006	July 1 1997 to December 31	1222 days with median of 33	PM_{10} from one of five monitoring sites	Mean daily PM_{10} = 16.11 μg/m^3, range =	Temperature, seasonality, day	hospitalization, respiratory	lower	

	2000, Brisbane Australia	patients per day admitted to hospital for respiratory disease		4.90 – 60.60	of week, long term trend, influenza			
Cooper et al. 1994	January 1994 Sydney fire (10 day event)	Data only shown in graphical form	hourly average scattering coefficient from a nephelometer used to distinguish before, during and after fires	Only shown in graphical form	None reported	acute asthma hospital presentations	higher	Periods compared had different days of week, did not control for temperature, and not enough information given on methods
Delfino et al. 2009	Southern California 2003	Unit of analysis is ZIP code-day. There were 45 days in the analysis, but does not state number of ZIP codes. Population covered was 20.5 million.	Zip code level $PM_{2.5}$ estimates from spatial interpolations from measured $PM_{2.5}$, light extinction, meteorological conditions and smoke information from MODIS satellite. Missing values were estimated from temporal profiles of continuous PM monitors at closely located sites or light extinction from visibility data, meteorological conditions and smoke info from MODIS. For nonfire periods, spatial interpolations using IDW, kriging or cokriging, but during fire polygons were created to represent the fire densities and $PM_{2.5}$ concentrations in each smoke-polygon were assigned.	During fires modeled mean $PM_{2.5}$ ranged from 42.1 to 76.1 μg/m^3	Temperature, relative humidity, pressure gradient, fungal spores (asthma only), income, age, race, gender, weekend, county	Hospitalizations for various respiratory and cardiovascular endpoints	lower	
Dennekamp et al. 2015	2006-2007 bushfire season Victoria, Australia	2046 out-of-hospital cardiac arrests	$PM_{2.5}$ from one monitoring station	IQR of $PM_{2.5}$ = 6.1 μg/m^3	Temperature, relative humidity, month, day of week, and hour of day.	out of hospital cardiac arrest	lower	
Duclos et al. 1990	August 1987, lightning fire in Northern	699 observed ER visits in 2.5 week fire period	temporal comparison of the fire period to two referent periods (one the previous month) and	Not reported	Seasonal and annual trends	emergency department visits and	moderate	Did not control for temperature or RH

	California		one in the previous year at the same time			hospitalizations for respiratory and mental health effects		
Elliott et al. 2013	British Columbia 2003-2010 during fire seasons (April1-September 30)	42456 LHA-days = (29 local health areas (LHAs) * 183 days per year * 8 years); average daily salbutamol dispensations ranged from 4.3 to 103.4 by LHA	$PM_{2.5}$ from one station per LHA, either the one nearest its centroid or its only one. For areas that didn't have $PM_{2.5}$ for the whole period, converted PM_{10} to $PM_{2.5}$ using regressions for the time period with both, or if no $PM_{2.5}$ then the regression from all of the other LHAs. Also dichotomized LHAs as fire affected by using MODIS fire pixels and chose the ones that were regularly impacted by fire.	Maximum concentrations of $PM_{2.5}$ in fire affected LHAs ranged 33.4 to 248.1 $\mu g/m^3$	Temperature, RH, year, month, and day of week	drug dispensations, salbutamol sulfate	lower	
Faustini et al. 2015	Ten cities in Spain, Italy, and Greece	20,087 study days across ten cities; daily mean natural deaths = 36	Smoky days versus non-smoky days classified from NAAP model (derived from AOD and fire plumes)	Smoky days PM_{10} ranged from 8-16 $\mu g/m^3$	Year, month, day of week, holidays, influenza, temperature, Saharan dust	Mortality	lower	
Haikerwal et al. 2015	2006-2007 wildfire episode in Victoria, Australia	457 out-of-hospital cardiac arrests; 2106 ED visits for IHD and 3274 hospital admissions for IHD	$PM_{2.5}$ modeled from a global chemical transport model dynamically downscaled using The Air Pollution Model	PM2.5 mean levels =15.43 $\mu g/m^3$ (IQR = 9.04 $\mu g/m^3$)	Time-stratified case control study controlled for day of week, seasons, time trends and individual covariates, also controlled for temperature and RH	Out-of hospital cardiac arrests, and hospitalizations and ED visits for IHD, acute MI, and angina	lower	
Hanigan et al. 2008	fire seasons, 1996-2005 Darwin, Australia	2410 days; 8279 hospital admissions	model of estimated exposure from visibility data	PM_{10} mean levels during fire period 21.2 ± 8.2 $\mu g/m^3$	RH, temperature, influenza, time trends, indigenous status, holidays	hospitalizations, cardiovascular	lower	

Henderson et al. 2011	July-September 2003 in British Columbia, fire season	281,711 people in cohort with 92 days of observation	(1) TEOM PM10 monitors, people assigned to nearest monitor to their postal address, (2) CALPUFF estimates of PM10 based on fire boundaries, and (3) binary smoke variable based on smoke boundaries from NOAAs fire detection tool: if there was a smoke plume over an area at any point during the day, it was considered exposed	PM_{10} mean levels 29.4 ± 30.7 μg/m^3	Temperature, day of week, week of study	Respiratory and cardiovascular hospitalizations and physician visits	lower	
Ho et al. 2014	2013 south Asian haze crisis	298 respondents	Self-report of perceived pollution standard index (PSI) as dangerous	Highest level pollution standard index of 401 on 0 to 500 scale.	None reported	Impact of Event Scale – Revised Survey, measure of psychological stress	higher	Self-report of exposure
Holstius et al. 2012	2003 Southern California Fires	886,034 births	temporal comparison of before, during and after fires	Not reported	Sex, gestational age, parity, maternal age, maternal education, maternal race, secular trend, season	birth weight	moderate	Not adjusted for maternal smoking
Ignotti et al. 2010	2004-2005 comparison of states in Brazilian Amazon	107 microregions	spatial comparison of % of annual hours with $PM_{2.5}$ > 80 μg/m^3	Assumed a threshold of 80 μg/m^3 based on Oregon standards	Human development index, a measure of education, earned income and longevity; and number of blood counts, an indicator of health service quality	hospitalization, respiratory	moderate	Did not control for meteorology/season and smoke prevalence
Jacobson et al. 2012	August to September 2006, Alta Floresta Brazil	309 children	$PM_{2.5}$ hourly measurements converted to 5-hour, 6-hour, 12-hour and 24-hour averages.	$PM_{2.5}$ mean levels = 24.34 ± 19.25 μg/m^3	age, height, weight, asthma status, passive smoking, use of	lung function	lower	

					medication, temperature, humidity, gender, occurrence of respiratory infections			
Jacobson et al. 2014	August to November 2008, Tangara da Serra, Brazil	234 children	PM_{10} and $PM_{2.5}$ and black carbon from one monitor at the school	PM_{10} mean levels = 62.7 ± 40.7 μg/m^3	Time trends, temperature, humidity	Lung function	lower	
Jalaludin et al. 2000	January 1994 Sydney	32 children for 31 days	PM_{10} from the monitor closest to each child's school	Not reported	Bushfire period, asthma medication usage, time trend, temperature, humidity, pollen counts, alternaria counts	lung function	moderate	Small sample size and duration
Jayachand ran 2009	1997 Southeast Asian Fires	67,454 subdistrict-months; average size of birth cohort 95.6 per subdistrict-month	Aerosol index from the Total Ozone Mapping Spectrometer (TOMS) by month interpolated to each spatial subdistrict	Not reported	Subdistrict population, fixed effects for subdistrict and for month, median log of food consumption, rainfall, predicted fertility, fuel use, health facilities	Birth cohort size	moderate	Coarse resolution exposure metric that may not have represented ground-level concentrations well
Johnston et al. 2002	Darwin April 1-October 31 2000, a period of minimal rainfall and	214 days; 256 total asthma presentations	PM_{10} averaged from two monitoring stations	Range of PM_{10} = 2.0 to 70 μg/m^3	Influenza, weekends	emergency department visits, asthma	moderate	Did not control for temperature

	almost continuous bushfire activity							
Johnston et al. 2006	seven month period in Darwin, Australia	251 people	$PM_{2.5}$ and PM_{10} from two monitors	PM_{10} mean levels 20 ±6.4 μg/m^3	Temperature, humidity, rainfall, pollen count, spore count, influenza rates, weekends, holidays, temporal autocorrelation	Asthma rescue medication usage; oral steroid medication usage	lower	
Johnston et al. 2007	Darwin, Australia three fire seasons, 2000, 2004, and 2005	2466 emergency admissions	PM_{10} from one monitoring station	Mean PM_{10} = 17.4 μg/m^{3,} range (1.1 to 70)	Day of week, month, year, influenza, temperature, humidity, rainfall, holidays	hospitalizations, asthma	lower	
Johnston et al. 2011	Sydney 1997-2004	284,326 deaths	Categorized days (high smoke days compared to non-smoke days). Days were classified as 'extreme events' based on if the PM_{10} city-wide average from 7 monitoring stations exceeded the 99th percentile for the time series (47.3ug/m3) and the cause of each event was verified to determine days which were due to smoke	Smoke days PM_{10} ranged from 47.3 – 114.8 μg/m^3	Day of week, month, year, influenza, temperature, humidity	mortality	lower	
Johnston et al. 2014	Sydney 1996-2004	630,000 ED presentations for respiratory conditions; 370,000 ED presentations for cardiovascular	Categorized days (high smoke days compared to non-smoke days). Days were classified as 'extreme events' based on if the PM_{10} city-wide average from 7 monitoring stations exceeded the 99th percentile for the time series (47.3μg/m^3) and the cause	Mean PM_{10} on smoke-affected days was 60.5 μg/m^3	Day of week, month, year, influenza, temperature, dew point, holiday	ED visits for respiratory and cardiovascular endpoints	lower	

		conditions	of each event was verified to determine days which were due to smoke					
Lee et al. 2009	Hoopa Valley Indian Reservation Fire of 1999	1882 clinic visits	One PM_{10} monitor and a comparison to the previous year	Weekly average PM_{10} levels ranged from 12.8 to 363.8 μg/m^3	Residence location (in or near reservation) and # of clinic visits in previous year (both done by stratification), age, sex	Respiratory and physician visits, respiratory	moderate	Did not control for temperature or humidity
Linares et al. 2014	Madrid days with advection from biomass burning from 2004-2009	2192 days	Effect of PM_{10} on mortality on days with advection of biomass burning	Mean PM_{10} on days with advection was 44.2 μg/m^3	Ozone, temperature, trend, seasonality	Mortality	lower	
Marshall et al. 2007	2003 Southern California Fires	357 respondents	self-reported difficulty breathing because of smoke or ashes	Not reported	Age, gender, race/ethnicity, education, employment status, income	PTSD or depression three months after fires	higher	Retrospective self-report of exposure
Martin et al. 2013	top 99% of days from 1994-2007 in Sydney, Newcastle and Wollongong	3,141,017 non-trauma hospital admissions in Sydney, 273,034 in Wollongong, and 345,736 in Newcastle	Categorized days (high smoke days compared to non-smoke days). Days were classified as 'extreme events' based on if the PM_{10} city-wide average from 7 monitoring stations exceeded the 99th percentile for the time series (47.3ug/m3) and the cause of each event was verified to determine days which were due to smoke	High smoke days Sydney PM_{10} = 67.3 μg/m^3, range = (47.3 to 114.8)	Day, month, year, temperature, humidity, dew point, influenza, holidays	Respiratory and cardiovascular hospitalization	lower	
McDermott et al. 2005	2003 Canberra, Australia wildfires	222 children	self-reported "saw smoke"	Not reported	None reported	post-traumatic stress disorder reaction index score and Strengths& Difficulties Score (based on	higher	Retrospective self-report of exposure

						emotional problems, conduct problems, and hyperactivity)		
Moore et al. 2006	British Columbia 2003 fires	Studied weekly rates of respiratory physician visits for six weeks in one year compared to ten previous years in two small communities. Population of Kelowna = 146,199. Population of Kamloops = 100,548.	temporal comparison; determined fire affected time periods by PM monitoring at each of two sites (Kelowna and Kamloops)	Graphics appear to demonstrate effects when $PM_{2.5}$ > 50 µg/m^3 only for Kelowna and not Kamloops	LHA population, seasonality by temporal comparison	physician visits for respiratory, cardiovascular or mental health endpoints	moderate	Small sample size, did not control for temperature
Morgan et al. 2010	daily exposure in Sydney 1994-2002	3103 days; average daily all-cause mortality = 56	PM_{10} from 8 monitoring locations, Defined bushfire days as days with city-wide 24hour average PM_{10} greater than the 99th percentile for the study period and verified with newspaper archives and other sources (note that could be bushfires or "fuel-reduction burns") -- and estimated background PM_{10} on bushfire days as the 30-day moving average of PM_{10} when bushfire days are set to missing	Bushfire days range of PM_{10} = 43-117 µg/m^3	Background PM10, temperature, humidity, time trend, day of week, influenza	Respiratory or cardiovascular hospitalization	lower	
Mott et al. 2002	1999 fire near Hoopa Valley National Indian Res, Aug 23-Nov3	289 interviews	temporal comparison	Not reported in tables	Stratified by time period	physician visits, respiratory	moderate	Self-reported outcomes, not adjusted for temperature

Mott et al. 2005	1997 Southeast Asian Fires	Monthly time-series of 35 months used to predict for five months of fire and compare to observed	temporal comparison	Not reported	Stratified by time period	Respiratory and cardiovascular hospitalizations	moderate	Short time series; did not control for temperature effects
Nunes et al. 2013	Brazilian Amazon 2005	107 microareas in the Brazilian Amazon	annual % of hours of $PM_{2.5}$ over 25 μg/m^3	Range of annual % of hours with $PM_{2.5}$ > 25 μg/m^3 = 0.00 – 43.89	controlled for human development index, family health unit, number of intensive care unit beds	Circulatory disease mortality	moderate	potentially insufficient control of regional differences related to mortality such as smoking prevalence
Prass et al. 2012	2001-2005 in Porto Velho, Brazil	60 months	Number of hot spots detected by the NOAA-12 satellite by month	61,154 hot spots over 5 year time period; number by month ranged from 0 to 8,775	Sex, year, month, season	Birth weight	higher	Did not control for temperature or other seasonally varying factors that relate to birth weight; exposure measurement may not relate to smoke exposure
Rappold et al. 2011	2008 peat bog fire in North Carolina, June 1-July14, 2008, but 10-12 June were considered the high exposure period	42 counties (18 exposed); 44 days with three considered high exposure days;	Temporal and regional comparison;<br>AOD to define exposed and unexposed counties, dichotomized to exposed if AOD >1.25 and then if >25% of county area was at AOD 1.25 or higher that day is exposed, but then a county was considered exposed if had 2 days in that exposure category; compared the high exposure days to non-exposure days for each county and then compared exposed to non-exposed counties	not reported	Day of week, stratified by age and sex. Although did not control for temperature, long-term trend or demographical differences between counties, authors note analyses that demonstrated that	Respiratory and cardiovascular ED visits	lower	

					confounding by these variables was not evident			
Resnick et al. 2015	2011 Wallow Fire Albuquerque, NM	Over all time periods there were 4525 cardiovascular ED visits and 4164 respiratory ED visits	Temporal comparison	Mean $PM_{2.5}$ during the fires=31.3 µg/m^3	None reported; stratified by sex and age and time period	Respiratory and cardiovascular ED visits	higher	Did not control for temperature, humidity, day of week, holidays or time trends
Sastry 2002	smoke from the 1997 fires of Indonesia in Malaysia, April-November 1997	52,742 deaths	PM_{10} for Kuala Lumpur for 1996-1997, used visibility data for other locations and other years	Mean daily PM_{10} = 64.2 ±43.0 µg/m^3. Range from 16.2 to 423.9 µg/m^3	Temperature, humidity, long-term trend, seasonality	mortality	lower	
Shaposhnikov et al. 2014	Moscow heat wave and wildfires, summer 2010	Time-series analysis from 2006-2010; Moscow averages about 300 deaths per day	City-average PM_{10}	Not reported	Long-term trend, seasonality, day of week, relative humidity, temperature as an interaction term	mortality	lower	
Smith et al. 1996	January 1994 western Sydney	Average daily asthma attendances at hospitals was 14.1 for control period and 10.7 for fire period	PM_{10} from three monitoring stations	Hourly PM_{10} ranged from 0.0 to 250.0 µg/m^3	Time period (controlled for year and season), temperature, humidity, wind speed, pressure, rainfaull, ozone, NO_2	emergency department visits, asthma	lower	
Tham et al. 2009	January to March 2003, Victoria, Australia	212 days; mean daily respiratory hospital	PM_{10} from one monitoring station in Melbourne, and two others in the Gippsland region of Victoria.	PM_{10} range of 0 to 289 µg/m^3	Day of week, time trend, temperature, humidity	Respiratory hospitalization and emergency department	lower	

		admissions = 48.43				visits		
Thelen et al. 2013	2007 San Diego, whole year including fire	121 days; mean daily ED visits=247.4	HYSPLIT air quality model was run with and without fire emissions estimates to get a way to quantify $PM_{2.5}$ just from wildfires.	Modeled $PM_{2.5}$ of wildfire origin range 0 to 403 $\mu g/m^3$, with corresponding range of RR of 1.0 to 1.41, but they do not give information to understand at what level of exposure the health effects become significant	Temperature, relative humidity, age groups, income categories, day of week	emergency department visits, respiratory	moderate	Did not control for long term trend or seasonality
Tse et al. 2015	Years before and after the 2003 and 2007 southern California wildfires	2195 asthmatic children for the 2003 fires and 2965 asthmatic children for the 2007 fires selected from an ongoing pediatric cohort	ZIP codes were classified as fire affected and not fire-affected, but the method for doing so was not explained in the paper	Not reported	Temporal trends accounted for in using data from a full year before and after	Physician-dispensed short-acting Beta agonists, physician-prescribed oral corticosteroids, ED visits and hospitalizations for asthma, newly diagnosed asthma	moderate	Method of classifying exposure was not made clear; no adjustment for other temporal changes that could affect asthma outcomes such as exposure to tobacco smoke, pollens, temperature
Vedal and Dutton 2006	2002 June Denver - two days, June 9 and June 18	Two days; daily average non-accidental mortality = 35.3	regional comparison	not reported	Investigates temperature and time but just descriptively, not statistically	mortality	higher	Very low power to detect an effect from just two days
Vora et al. 2011	San Diego 2007 5 day firestorm	8 subjects followed for 3 periods of four days	Temporal comparison	Mean morning $PM_{2.5}$ = 71.8 ± 24.5 $\mu g/m^3$	Time periods	lung function and # of rescue medication doses used	moderate	Small sample size and did not control for temperature or humidity or exposure to environmental tobacco smoke
Wiwatana date & Liwsrisak	Chiang Mai, Thailand, August 15,	121 asthmatic subjects followed for	Air quality monitor in city center	$PM_{2.5}$ ranged from 13.19 $\mu g/m^3$to 223.83 $\mu g/m^3$	gender, age, asthma severity, day of week,	Lung function	moderate	Multipollutant models that did not deal with

un 2011	2005 to June 30, 2006	306 days			weight, pressure, temperature, sunshine duration, rain quantity and random effects			collinearity; did not adjust for time trends or seasonality
Yao et al. 2014	British Columbia 2003-2010 fire seasons	89 local health areas; total population over 4 million, April through September for ten years	PM monitoring data for 29 local health areas; modeled PM2.5 from a combination of AOD from MODIS, sum of fire radiative power from MODIS hot spots, and hand drawn smoke plumes from the NOAA Hazard Mapping System for all 89 local health areas	Mean daily measured $PM_{2.5}$ was 5.9±5.2 μg/m^3; Mean daily measured $PM_{2.5}$ on extreme fire days was 10.2±11.1 μg/m^3	Temperature, temporal trends	Dispensations of salbutamol and nitroglycerin; physician visits for asthma, upper respiratory infections, lower respiratory infections, otitis media and all cardiovascular diseases	lower	

Table S2: Effect estimates for original epidemiological research studies (N=53), regardless of level of potential bias, ordered by health outcome and type of effect estimate.

Article	Outcome	Lag	Type of Effect Estimate	Effect Estimate	Comment
Mortality, all-cause					
Sastry 2002	mortality, all-cause	one day	RR per 10 μg/m^3 PM_{10}	1.19 (0.98 , 1.41)	
Morgan et al. 2010	mortality, all-cause	one day	RR per 10 μg/m^3 PM_{10}	1.01 (1.00 , 1.02)	derived from reported percent increase; only best lag is reported here
Johnston et al. 2011	mortality, all-cause	one day	OR high smoke versus non-smoke days	1.05 (1.00 , 1.10)	
Faustini et al. 2015	Mortality, natural	0-1 day	RR smoky versus non-smoky days	1.02 (0.99, 1.05)	Derived from reported percent increase
Linares et al. 2014	Mortality, natural	Lag 2	RR per 10 μg/m^3 PM_{10}	1.035 (1.011, 1.060)	
Shaposhnikov et al. 2014	Mortality, non-accidental	Lags 0-6 cumulative	RR per 10 μg/m^3 PM_{10} at different levels of temperature	1.004 (1.001 – 1.008) at T <18°C<br>1.008 (1.004 – 1.011) at T=22°C<br>1.014 (1.010 – 1.019) at T=>30°C	Derived from reported percent increase
Analitis et al. 2011	mortality, all-cause	same day	RR large fire versus no fire days	1.50 (1.37 , 1.63)	derived from reported percent increase
Mortality, respiratory					
Analitis et al. 2011	mortality, respiratory	same day	RR large fire versus no fire days	1.92 (1.48 , 2.50)	derived from reported percent increase
Johnston et al. 2011	mortality, respiratory	one day lag	OR high smoke versus non-smoke days	1.09 (0.88 , 1.36)	

Morgan et al. 2010	mortality, respiratory	same day	RR per 10 μg/m^3 PM_{10}	1.00 (0.97 , 1.04)	derived from reported percent increase; only best lag is reported here
Faustini et al. 2015	Mortality, respiratory	0-5	RR smoky versus non-smoky days	0.97 (0.90, 1.03)	Derived from reported percent increase
Linares et al. 2014	Mortality, respiratory	Lag 2	RR per 10 μg/m^3 PM_{10}	No effect reported because it was not statistically significant	
Mortality, cardiovascular					
Analitis et al. 2011	mortality, cardiovascular	same day	RR large fire versus no fire days	1.61 (1.43 , 1.80)	derived from reported percent increase
Johnston et al. 2011	mortality, cardiovascular	one day lag	OR high smoke versus non-smoke days	1.07 (0.98 , 1.18)	
Morgan et al. 2010	mortality, cardiovascular	same day	RR per 10 μg/m^3 PM_{10}	1.01 (0.99 , 1.02)	derived from reported percent increase; only best lag is reported here
Nunes et al. 2013	Mortality, cardiovascular in people 65 years of age and older	NA (cross-sectional comparison)	RR for one unit increase in annual percentage of hours greater than 25 μg/m^3 $PM_{2.5}$	1.01 (p-value reported as 0.035)	Derived from adjusted beta coefficient from multiple linear regression
Faustini et al. 2015	Mortality, circulatory	0-5	RR smoky versus non-smoky days	1.06 (1.10, 1.12)	Derived from reported percent increase
Linares et al. 2014	Mortality, circulatory	Lag 2	RR per 10 μg/m^3 PM_{10}	No effect reported for PM_{10} because it was not statistically significant	
Lung function					
Jacobson et al. 2012	lung function	same day	change in peak expiratory flow (liters/minute) for non-asthmatics associated with $PM_{2.5}$	-0.38 (-0.62 , -0.14)	

Jacobson et al. 2014	Lung function	Lag 3	change in peak expiratory flow (liters/minute) for all children regardless of asthma status with PM_{10}	-0.25 (-0.40, -0.10)	Presented results for all children, but effects were strongest among youngest. Investigated many lags, only presented one here.
Jalaludin et al. 2000	lung function	same day	change in peak expiratory flow rate - children without bronchial hyper-reactivity	-1.03 (-1.95 , -0.11)	calculated from beta and SE - assumed linear model per unit change in PM_{10} based on what was presented in the paper
Respiratory morbidity, all					
Lee et al. 2009	physician visits, respiratory		OR per 10 μg/m^3 PM_{10}	1.77 (1.51 , 2.09)	this RR is for a unit change in the log of PM_{10}
Henderson et al. 2011	physician visits, respiratory	same day	OR per 10 μg/m^3 PM_{10}	1.02 (1.01 , 1.03)	presented results are associated with monitored values of PM. Similar results were found using modeled and remotely sensed estimates of smoke exposure.
Moore et al. 2006	physician visits, respiratory		observed compared to 10-year mean	46-78% increase over 10-year mean rates	
Mott et al. 2002	physician visits, respiratory		percent increase in fire year compared to percent increase in non-fire year	11.9% (10.4-13.4) increase in fire year and 8.9% (7.5-10.3) expected from previous year in September, 19.2%(17.2-21.3)in fire year compared to 10.7% (9.1-12.3) increase in previous year	
Lee et al. 2009	physician visits, all respiratory		OR per 10 μg/m^3 PM_{10}	1.36 (1.24 , 1.50)	this RR is for a unit change in the log of PM_{10}

Rappold et al. 2011	emergency department visits, respiratory	lag0-5 cumulative	RR comparing fire period to reference period	1.66 (1.38 , 1.99)	results presented here are for smoke-affected counties only
Tham et al. 2009	emergency department visits, respiratory	same day	RR per 10 μg/m^3 PM_{10}	1.01 (1.00 , 1.02)	*calculated from 25th-75th range to 10 μg/ m^3
Thelen et al. 2013	emergency department visits, respiratory	cumulative lag exposure kernel centered at same day and with SD of 1 day	OR per 10 μg/m^3 wildfire PM	1.00 (1.00 , 1.01)	original estimates were per unit μg/m^3
Johnston et al. 2014	ED visits, respiratory	Lag 0	OR comparing smoke days to non-smoke days	1.07 (1.04, 1.10)	
Resnick et al. 2015	ED visits, respiratory	NA	RR comparing fire period to pre-fire period	0.83 (0.77, 0.90)	
Tham et al. 2009	hospitalization, respiratory	same day	RR per 10 μg/m^3 PM_{10}	1.00 (0.99 , 1.01)	calculated from 25th-75th range to 10 μg/m^3
Morgan et al. 2010	hospitalization, respiratory	same day	RR per 10 μg/m^3 PM_{10}	1.01 (1.00 , 1.02)	derived from reported percent increase; only best lag is reported here
Henderson et al. 2011	hospitalization, respiratory	same day	OR per 10 μg/m^3 PM_{10}	1.05 (1.00, 1.10)	*only presenting here results associated with monitored values of PM. Similar results were found using modeled and remotely sensed

					estimates of smoke exposure.
Johnston et al. 2007	hospitalization, respiratory	same day	OR per 10 μg/m^3 PM_{10}	1.08 (0.98 , 1.18)	for whole population
Delfino et al. 2009	hospitalization, respiratory	2-day moving average	RR per 10 μg/m^3 $PM_{2.5}$	1.03 (1.01 , 1.04)	This estimate is for the fire period; paper includes estimates for pre-fire and post-fire periods also
Martin et al. 2013	hospitalization, respiratory	same day	OR for high smoke days compared to non-smoke days	1.05 (1.02 , 1.09)	here only reporting the best lag result for Sydney, not other cities
Chen et al. 2006	hospitalization, respiratory	same day	RR comparing highest exposure category (>20 μg/m^3) against the lowest category (<15 μg/m^3), for the bushfire period	1.19 (1.09 , 1.30)	comparing highest exposure category (>20 μg/m^3) against the lowest category (<15 μg/m^3), for the bushfire period
Cancado et al. 2006	hospitalization, respiratory		RR for biomass burning factor from factor analysis	1.52 (1.12, 2.04)	for elderly only; calculated from effect estimate and SE non-exponentiated
Mott et al. 2005	hospitalization, respiratory	NA	observed compared to CI of expected	184 observed and 89.3-174.0 expected	all ages
Ignotti et al. 2010	hospitalization, respiratory		increase in respiratory hospitalizations associated with % annual hours > 80 μg/m^3	0.052 increase (p-value=0.017)	ecological analysis only
Asthma, exacerbations					

Jacobson et al. 2012	lung function	same day	change in peak expiratory flow for asthmatics	-0.18 (-0.66 , 0.31)	
Jalaludin et al. 2000	lung function	same day	change in peak expiratory flow rate - all children	-0.09 (-1.17 , 0.98)	calculated from beta and SE - assumed linear model per unit change in PM_{10} based on what was presented in the paper
Vora et al. 2011	lung function		difference between fires and non-fires	p-values ranged from 0.35 to 0.80 for different lung function metrics	only p-values reported
Wiwatandate & Liwsrisakun 2011	lung function	lag 6	change in peak expiratory flow rate among asthmatic people over age 12	-0.01 (-0.01, 0.00)	Lag 5 was also significant for PM_{10}
Elliott et al. 2013	drug dispensations, salbutamol sulfate	Same day	RR per 10 $\mu g/m^3$ $PM_{2.5}$	1.06 (1.04 , 1.07)	*these dispensations are for both asthma and COPD, but are placed in the asthma section of this table
Yao et al. 2014	drug dispensations, salbutamol sulfate	Mean of same day and previous day	RR per 10 $\mu g/m^3$ $PM_{2.5}$	1.04 (1.03 – 1.06)	Estimate from modeled $PM_{2.5}$; similar results for modeled $PM_{2.5}$
Tse et al. 2015	Physician-dispensed Beta-agonists	NA	Compared total for year after fires to year before fires	$p < 0.05$	
Tse et al. 2015	Physician-prescribed oral coriticosteroids	NA	Compared total for year after fires to year before fires	$p >= 0.05$	
Arbex et al. 2000	Hospital visits for inhalation therapy	Moving average of days 1-5	RR per 10 mg sediment weight	1.09 (1.00 – 1.19)	

Caamano-Isorna et al. 2011	drug dispensations for obstructive airway diseases		high exposure regions post-fire compared to no exposure regions pre-fire	1.18 (1.01, 1.35)	calculated from percent increase; presenting only results for male pensioners, also sig increase for women pensioners; *these dispensations are for both asthma and COPD, but are placed in the asthma section of this table
Vora et al. 2011	# of rescue medication doses used		only significance values presented for difference between fires and non-fires	p=0.03	
Johnston et al. 2006	rescue medication usage	one day	OR per 10 μg/m^3 PM_{10}	1.01 (0.99, 1.04)	
Johnston et al. 2006	oral steroid medication usage	one day	OR per 10 μg/m^3 PM_{10}	1.54 (1.01, 2.34)	
Henderson et al. 2011	physician visits, asthma	same day	OR per 10 μg/m^3 PM_{10}	1.06 (1.03 , 1.11)	*only presenting here results associated with monitored values of PM. Similar results were found using modeled and remotely sensed estimates of smoke exposure.
Yao et al. 2014	physician visits, asthma	Mean of same day and previous day	RR per 10 μg/m^3 $PM_{2.5}$	1.06 (1.04 – 1.08)	Estimate from modeled $PM_{2.5}$; similar results for modeled $PM_{2.5}$

Johnston et al. 2002	emergency department visits, asthma	Same day	RR per 10 μg/m^3 PM_{10}	1.20 (1.09 , 1.34)	
Rappold et al. 2011	emergency department visits, asthma	Lag 0-5 cumulative	RR comparing fire period to reference period	1.65 (1.25 , 2.17)	results presented here are for smoke-affected counties only; see paper for comparison to non-smoke affected counties
Duclos et al. 1990	emergency department visits, asthma	NA	observed/expected	1.4 (p-value<0.001)	
Smith et al. 1996	emergency department visits, asthma		difference in difference calculation	0.0067 (-0.0007, 0.0141)	temporal comparison of week of fire to same week a year before - presented difference in proportion of all visits that were for asthma for fire weeks compared to previous year minus the same difference for weeks surrounding the fire of both years and found no significant effect
Johnston et al. 2014	ED visits, asthma	Lag 0	OR comparing smoke days to non-smoke days	1.23 (1.15, 1.30)	
Resnick et al. 2015	ED visits, asthma	NA	RR comparing fire period to pre-fire period	1.73 (1.03-2.77)	this estimate is for ages 65+, non-significant findings for other ages; also found higher effects on women than men

					for asthma
Tse et al. 2015	ED visits, asthma among children with asthma	NA	Compared total for year after fires to year before fires	$p >= 0.05$	
Morgan et al. 2010	hospitalizations, asthma	same day	RR per 10 µg/m^3 PM_{10}	1.05 (1.02 , 1.08)	15-64 year-olds; derived from reported percent increase; only best lag is reported here
Johnston et al. 2007	hospitalizations, asthma	same day	OR per 10 µg/m^3 PM_{10}	1.14 (0.90 , 1.44)	for whole population
Delfino et al. 2009	hospitalizations, asthma	2-day moving average	RR per 10 µg/m^3 $PM_{2.5}$	1.05 (1.02 , 1.08)	This estimate is for the fire period; paper includes estimates for pre-fire and post-fire periods also
Arbex et al. 2007	hospitalizations, asthma	5-day moving average	RR per 10 units of TSP	1.12 (1.05 , 1.18)	calculated from percentage increase
Martin et al. 2013	hospitalizations, asthma	same day	OR for high smoke days compared to non-smoke days	1.12 (1.05 , 1.19)	here only reporting the best lag result for Sydney, not other cities
Tse et al. 2015	hospitalizations, asthma among children with asthma	NA	Compared total for year after fires to year before fires	$p >= 0.05$	
Asthma, new diagnoses					
Tse et al. 2015	newly diagnosed asthma	NA	Compared total for year after fires to year before fires	Decline in new asthma diagnoses post-fire ($p < 0.05$)	

Chronic obstructive pulmonary disease (exacerbations)					
Yao et al. 2014	physician visits, COPD	Mean of same day and previous day	RR per 10 µg/m^3 $PM_{2.5}$	1.02 (1.00 – 1.03)	Estimate from modeled $PM_{2.5}$; similar results for modeled $PM_{2.5}$
Rappold et al. 2011	emergency department visits, COPD	Lag 0-5 cumulative	RR comparing fire period to reference period	1.73 (1.06 , 2.83)	results presented here are for smoke-affected counties only; see paper for comparison to non-smoke affected counties
Duclos et al. 1990	emergency department visits, COPD	NA	observed/expected	1.3 (p-value =0.02)	
Johnston et al. 2014	ED visits, COPD	Lag 0	OR comparing smoke days to non-smoke days	1.12 (1.02, 1.24)	
Morgan et al. 2010	hospitalizations, COPD	lag 2	RR per 10 µg/m^3 PM_{10}	1.04 (1.01 , 1.06)	Only analyzed COPD for 65+; similar findings for lags 0 through 3, but presented largest finding here at lag 2; derived from reported percent increase; only best lag is reported here
Johnston et al. 2007	hospitalizations, COPD	same day	OR per 10 µg/m^3 PM_{10}	1.21 (1.00 , 1.47)	for whole population; 1.98 (1.10,3.59) for Indigenous
Delfino et al. 2009	hospitalizations, COPD	2-day moving average	RR per 10 µg/m^3 $PM_{2.5}$	1.04 (1.00 , 1.08)	Ages 20-99; This estimate is for the fire period; paper includes estimates for pre-fire and post-fire

					periods also
Martin et al. 2013	hospitalizations, COPD	same day	OR for high smoke days compared to non-smoke days	1.13 (1.05 , 1.22)	here only reporting the best lag result for Sydney, not other cities
Mott et al. 2005	hospitalizations, COPD	NA	observed compared to CI of expected	255 observed, 152.4-250.2 expected	all ages
Respiratory infections					
Henderson et al. 2011	Physician visits, acute upper respiratory infections	same day	OR per 10 µg/m^3 PM_{10}	0.99 (0.47 , 1.98)	Calculated from effect found for 30 unit change in PM_{10}; *only presenting here results associated with monitored values of PM. Similar results were found using modeled and remotely sensed estimates of smoke exposure.
Yao et al. 2014	physician visits, upper respiratory infections	Mean of same day and previous day	RR per 10 µg/m^3 $PM_{2.5}$	1.03 (1.02 – 1.05)	Estimate from measured $PM_{2.5}$; results from modeled $PM_{2.5}$ was null and not reported in tabular form.
Yao et al. 2014	physician visits, lower respiratory infections	Mean of same day and previous day	RR per 10 µg/m^3 $PM_{2.5}$	1.03 (1.00 – 1.05)	Estimate from modeled $PM_{2.5}$; similar results for modeled $PM_{2.5}$
Rappold et al. 2011	emergency department visits, upper respiratory infections	Lag 0-5 cumulative	RR comparing fire period to reference period	1.68 (0.94 , 3.00)	results presented here are for smoke-affected counties only; see paper for

					comparison to non-smoke affected counties
Duclos et al. 1990	hospitalizations, upper respiratory infections	NA	observed/expected	1.5 (p-value<0.001)	
Johnston et al. 2007	hospitalizations, upper respiratory infections		OR per 10 μg/m^3 PM_{10}	Effect Estimate not reported.	
Pneumonia and bronchitis					
Rappold et al. 2011	ED visits for pneumonia and acute bronchitis	Lag 0-5 cumulative	RR comparing fire period to reference period	1.59 (1.07 , 2.34)	results presented here are for smoke-affected counties only; see paper for comparison to non-smoke affected counties
Johnston et al. 2014	ED visits, pneumonia and bronchitis	Lag 0	OR comparing smoke days to non-smoke days	1.02 (0.95, 1.10)	
Delfino et al. 2009	hospitalizations for acute bronchitis and bronchiolitis	2-day moving average	RR per 10 μg/m^3 $PM_{2.5}$	1.10 (1.02 , 1.18)	Acute bronchitis and bronchiolitis; This estimate is for the fire period; paper includes estimates for pre-fire and post-fire periods also
Delfino et al. 2009	hospitalizations for pneumonia	2-day moving average	RR per 10 μg/m^3 $PM_{2.5}$	1.03 (1.01, 1.05)	Pneumonia; This estimate is for the fire period; paper includes estimates for pre-fire and post-fire periods also

Morgan et al. 2010	hospitalizations for pneumonia and acute bronchitis	lag 1	RR per 10 µg/m^3 PM_{10}	1.03 (1.02 , 1.06)	pneumonia and acute bronchitis for 65+ attributable to bushfire days; derived from reported percent increase; only best lag is reported here
Martin et al. 2013	hospitalizations for pneumonia and acute bronchitis	lag 2	OR for high smoke days compared to non-smoke days	1.26 (1.03, 1.55)	best lag for Newcastle; non-significant findings for Sydney and Wollongong
Duclos et al. 1990	hospitalizations for bronchitis	NA	observed/expected	1.2 (p-value = 0.03)	bronchitis
Duclos et al. 1990	hospitalizations for pneumonia	NA	observed/expected	1.0 (p-value = 0.4)	pneumonia
Cardiovascular disease, all					
Yao et al. 2014	Dispensations of fast-acting nitroglycerin for angina	Mean of same day and previous day	RR per 10 µg/m^3 $PM_{2.5}$	1.03 (1.01 – 1.05)	Effect for extreme fire days; RR was null for all days
Henderson et al. 2011	physician visits, cardiovascular	same day	OR per 10 µg/m^3 PM_{10}	1.00 (0.99 , 1.01)	*only presenting here results associated with monitored values of PM. Similar results were found using modeled and remotely sensed estimates of smoke exposure.
Moore et al. 2006	physician visits, cardiovascular			data not shown	

Lee et al. 2009	physician visits, all circulatory illness		OR per 10 μg/m^3 PM_{10}	1.13 (0.94 , 1.37)	this RR is for a unit change in the log of PM_{10}
Yao et al. 2014	physician visits, cardiovascular	Mean of same day and previous day	RR per 10 μg/m^3 $PM_{2.5}$	Null; data only shown graphically	
Rappold et al. 2011	emergency department visits, cardiovascular	Lag 0-5 cumulative	RR comparing fire period to reference period	1.13 (0.95 , 1.35)	results presented here are for smoke-affected counties only; see paper for comparison to non-smoke affected counties
Johnston et al. 2014	ED visits, COPD	Lag 0	OR comparing smoke days to non-smoke days	1.00 (0.96, 1.04)	
Morgan et al. 2010	hospitalizations, cardiovascular	lag 2	RR per 10 μg/m^3 PM_{10}	1.01 (0.99 , 1.01)	derived from reported percent increase; only best lag is reported here
Hanigan et al. 2008	hospitalizations, cardiovascular	same day	RR per 10 μg/m^3 PM_{10}	0.97 (0.91 , 1.02)	
Henderson et al. 2011	hospitalizations, cardiovascular	same day	OR per 10 μg/m^3 PM_{10}	1.00 (0.96 , 1.05)	*only presenting here results associated with monitored values of PM. Similar results were found using modeled and remotely sensed estimates of smoke exposure.
Johnston et al. 2007	hospitalizations, cardiovascular		OR per 10 μg/m^3 PM_{10}	data not shown	

Martin et al. 2013	hospitalizations, cardiovascular		OR for high smoke days compared to non-smoke days	data not shown	
Resnick et al. 2015	ED visits, all cardiovascular	NA	RR comparing fire period to pre-fire period	1.08 (1.00, 1.16)	
Congestive Heart Failure					
Rappold et al. 2011	emergency department visits, congestive heart failure	Lag 0-5 cumulative	RR comparing fire period to reference period	1.37 (1.01 , 1.85)	results presented here are for smoke-affected counties only; see paper for comparison to non-smoke affected counties
Morgan et al. 2010	hospitalizations, congestive heart failure	lag 2	RR per 10 μg/m^3 PM_{10}	1.00 (0.99 , 1.01)	derived from reported percent increase; only best lag is reported here
Delfino et al. 2009	hospitalizations, congestive heart failure	2-day moving average	RR per 10 μg/m^3 $PM_{2.5}$	1.02 (0.99 , 1.04)	This estimate is for the fire period; paper includes estimates for pre-fire and post-fire periods also
Martin et al. 2013	hospitalizations, congestive heart failure	lag 3	OR for high smoke days compared to non-smoke days	1.05 (0.96 , 1.14)	here only reporting the best lag result for Sydney, not other cities
Cardiac Failure					
Dennekamp et al. 2015	out of hospital cardiac arrest	48-hour	OR per 10 μg/m^3 $PM_{2.5}$	1.04 (1.00 , 1.08)	OR derived from reported percent increase in IQR $PM_{2.5}$
Johnston et al. 2014	ED visits, Cardiac failure	Lag 0	OR comparing smoke days to non-smoke days	1.05 (0.95, 1.17)	
Ischemic heart disease					
Johnston et al. 2014	ED visits, Ischemic heart disease	Lag 2	OR comparing smoke days to non-smoke days	1.07 (1.00, 1.15)	Non-significant at other lags (0,1, and 3 days)

Morgan et al. 2010	hospitalizations, ischemic heart disease	same day	RR per 10 μg/m^3 PM_{10}	1.00 (0.99 , 1.02)	derived from reported percent increase; only best lag is reported here
Delfino et al. 2009	hospitalizations, ischemic heart disease	2-day moving average	RR per 10 μg/m^3 $PM_{2.5}$	1.01 (0.99 , 1.02)	This estimate is for the fire period; paper includes estimates for pre-fire and post-fire periods also
Johnston et al. 2007	hospitalizations, ischemic heart disease	same day	OR per 10 μg/m^3 PM_{10}	0.82 (0.68 , 0.98)	for whole population; 1.71 (1.14,2.55) for Indigenous population
Martin et al. 2013	hospitalizations, ischemic heart disease	lag 2	OR for high smoke days compared to non-smoke days	1.03 (0.98 , 1.08)	here only reporting the best lag result for Sydney, not other cities
Mott et al. 2005	hospitalizations, ischemic heart disease	NA	observed compared to CI of expected	109 observed when 51.5-91.5 expected	results for ages 19-39 only significant age group
Lee et al. 2009	physician visits, coronary artery disease		OR per 10 μg/m^3 PM_{10}	1.48 (1.11 , 1.97)	this RR is for a unit change in the log of PM_{10}
Resnick et al. 2015	ED visits, ischemic heart disease	NA	RR comparing fire period to pre-fire period	1.17 (0.89, 1.55)	
Hypertension					
Henderson et al. 2011	physician visits, hypertension	same day	OR per 10μg/m^3 PM_{10}	1.00 (0.98 , 1.01)	Calculated from effect found for 30 unit change in PM_{10}; *only presenting here results associated with monitored values of PM. Similar results were found using modeled and remotely sensed estimates of smoke exposure.

Arbex et al. 2010	hospitalizations, hypertension	3-day moving average	RR per 10 μg/m^3 TSP	1.13 (1.06 , 1.20)	burning season estimate was 30% higher than non-burning season; calculated from percent increase
Resnick et al. 2015	ED visits, hypertensive disease	NA	RR comparing fire period to pre-fire period	1.08 (0.97, 1.20)	
Cardiac dysrhythmias					
Johnston et al. 2014	ED visits, arrhythmias	Lag 0	OR comparing smoke days to non-smoke days	0.97 (0.89, 1.06)	
Delfino et al. 2009	hospitalizations, dysrhythmias	2-day moving average	RR per 10 μg/m^3 $PM_{2.5}$	0.99 (0.96 , 1.02)	This estimate is for the fire period; paper includes estimates for pre-fire and post-fire periods also
Martin et al. 2013	hospitalizations, arrhythmia	lag 2	OR for high smoke days compared to non-smoke days	0.96 (0.88 , 1.04)	here only reporting the best lag result for Sydney, not other cities
Cerebrovascular disease					
Johnston et al. 2014	ED visits, cerebrovascular disease	Lag 0	OR comparing smoke days to non-smoke days	0.99 (0.91, 1.08)	
Resnick et al. 2015	ED visits, cerebrovascular disease	NA	RR comparing fire period to pre-fire period	1.69 (1.03, 2.77)	This estimate is just for ages 20-64; non-significant findings for 65+ and for 0-19
Delfino et al. 2009	hospitalizations, cerebrovascular disease and stroke	2-day moving average	RR per 10 μg/m^3 $PM_{2.5}$	1.02 (1.00 , 1.04)	This estimate is for the fire period; paper includes estimates for pre-fire and post-fire periods also

Morgan et al. 2010	hospitalizations, stroke	lag 2	RR per 10 μg/m^3 PM_{10}	1.01 (0.99 , 1.03)	just stroke, converted from percentage increase
Birth outcomes					
Holstius et al. 2012	birth weight	NA	decline in birth weight associated with gestation during fires compared to gestation not during fires	7.0 g lower [95% confidence interval (CI): –11.8, –2.2]	only presenting results for full pregnancy, not divided by trimester
Breton et al. 2011	birth weight	NA		not yet published	these findings have not yet been published, therefore we cannot publish the estimates
Jayachandran 2009	cohort size	NA	proportion of cohort surviving compared to normal cohort due to exposure to fire smoke during last three months of pregnancy	0.97 (0.94, 0.99)	calculated from log effect estimate and SE
Candido da Silva et al. 2014	Low birth weight	NA	OR of low birth weight associated with PM2.5 during second and third trimester for highest exposed quartile compared to lowest exposed quartile	1.51 (1.04, 2.17)	*Only presented second trimester results
Prass et al. 2012	Birth weight	NA	effect of monthly number of satellite detected hot spots on mean monthly birth weight in boys	-0.004485, p-value = 0.0431	Did not find an effect of monthly hot spots on monthly birth weight for girls
Mental Health					
McDermott et al. 2005	post-traumatic stress disorder reaction index score	NA	t-test for comparing scores for those who reported seeing smoke to those who reported not seeing smoke	t=1.63, p=0.11	p-value calculated from reported t-test and degrees of freedom

McDermott et al. 2005	Strengths& Difficulties Score (based on emotional problems, conduct problems, and hyperactivity)	NA	t-test for comparing scores for those who reported seeing smoke to those who reported not seeing smoke	t=3.76, p=0.0003	p-value calculated from reported t-test and degrees of freedom
Marshall et al. 2007	PTSD or depression three months after fires	NA	OR for those who reported difficulty breathing because of fires compared to those who did not	2.09 (1.10, 3.98)	
Caamano-Isorna et al. 2011	drug dispensations for anxiolytics	NA	high exposure regions post-fire compared to no exposure regions pre-fire	1.21 (1.10, 1.33)	calculated from percent increase; presenting only results for male pensioners, also sig increase for male non-pensioners; only significant for medium exposure regions compared to non-exposed
Moore et al. 2006	physician visits, mental illness	NA		data not shown	
Duclos et al. 1990	hospitalizations, mental health	NA	observed/expected	1.1 (p-value=0.4)	
Ho et al. 2014	Impact of Event Scale – Revised Survey, measure of psychological stress	NA	Chi-squared	Those who perceived lower PSI values as dangerous were more likely to have higher IES-R stress values (p = 0.047)	

*effect estimates for symptoms are not included in this table because of their varied nature.

References

Analitis A, Georgiadis I, Katsouyanni K. 2012. Forest fires are associated with elevated mortality in a dense urban setting. Occup Environ Med 69:158-162.

Arbex MA, Bohm GM, Saldiva PH, Conceicao GM, Pope AC 3rd, Braga AL. 2000. Assessment of the effects of sugar cane plantation burning on daily counts of inhalation therapy. J Air Waste Manag Assoc (1995) 50:1745-1749.

Arbex MA, Martins LC, de Oliveira RC, Pereira LAA, Arbex FF, Cancado JED, et al. 2007. Air pollution from biomass burning and asthma hospital admissions in a sugar cane plantation area in Brazil. J Epidemiol Community Health 61:395-400.

Arbex MA, Saldiva PHN, Pereira LAA, Braga ALF. 2010. Impact of outdoor biomass air pollution on hypertension hospital admissions. J Epidemiol Community Health 64:573-579.

Azevedo JM, Goncalves FL, de Fatima Andrade M. 2011. Long-range ozone transport and its impact on respiratory and cardiovascular health in the north of Portugal. Int J Biometeorol 55:187-202.

Brook RD, Rajagopalan S, Pope CA 3rd, Brook JR, Bhatnagar A, Diez-Roux AV, et al. 2010. Particulate matter air pollution and cardiovascular disease: An update to the scientific statement from the American Heart Association. Circulation 121:2331-2378.

Caamano-Isorna F, Figueiras A, Sastre I, Montes-Martinez A, Taracido M, Pineiro-Lamas M. 2011. Respiratory and mental health effects of wildfires: An ecological study in Galician municipalities (north-west Spain). Environ health 10:48.

Cancado JE, Saldiva PHN, Pereira LAA, Lara L, Artaxo P, Martinelli LA, et al. 2006. The impact of sugar cane-burning emissions on the respiratory system of children and the elderly. Environ Health Perspect 114:725-729.

Candido da Silva AM, Moi GP, Mattos IE, Hacon Sde S. 2014. Low birth weight at term and the presence of fine particulate matter and carbon monoxide in the Brazilian Amazon: A population-based retrospective cohort study. BMC Pregnancy Childbirth 14:309.

Chen L, Verrall K, Tong S. 2006. Air particulate pollution due to bushfires and respiratory hospital admissions in Brisbane, Australia. Int J Environ Res Public Health 16:181-191.

Cooper CW, Mira M, Danforth M, Abraham K, Fasher B, Bolton P. 1994. Acute exacerbations of asthma and bushfires. Lancet 343:1509.

Delfino RJ, Brummel S, Wu J, Stern H, Ostro B, Lipsett M, et al. 2009. The relationship of respiratory and cardiovascular hospital admissions to the southern California wildfires of 2003. Occup Environ Med 66:189-197.

Delfino RJ, Staimer N, Tjoa T, Arhami M, Polidori A, Gillen DL, et al. 2010. Associations of primary and secondary organic aerosols with airway and systemic inflammation in an elderly panel cohort. Epidemiol 21:892-902.

Dennekamp M, Abramson MJ. 2011. The effects of bushfire smoke on respiratory health. Respirology 16:198-209.

Dennekamp M, Straney LD, Erbas B, Abramson MJ, Keywood M, Smith K, et al. 2015. Forest fire smoke exposures and out-of-hospital cardiac arrests in Melbourne, Australia: A case-crossover study. Environ Health Perspect. doi:10.1289/ehp.1408436

Duclos P, Sanderson LM, Lipsett M. 1990. The 1987 forest fire disaster in California: Assessment of emergency room visits. Arch Environ Health 45:53-58.

Elliott CT, Henderson SB, Wan V. 2013. Time series analysis of fine particulate matter and asthma reliever dispensations in populations affected by forest fires. Environ Health 12:11.

Faustini A, Alessandrini ER, Pey J, Perez N, Samoli E, Querol X, et al. 2015. Short-term effects of particulate matter on mortality during forest fires in southern Europe: Results of the MED-PARTICLES project. Occup Environ Med 72:323-329.

Finlay SE, Moffat A, Gazzard R, Baker D, Murray V. 2012. Health impacts of wildfires. PLoS currents 4:e4f959951cce959952c.

Flannigan M, Cantin AS, de Groot WJ, Wotton M, Newbery A, Gowman LM. 2013. Global wildland fire season severity in the 21st century. Forest Ecol Manag 294:54-61.

Flannigan MD, Krawchuk MA, de Groot WJ, Wotton BM, Gowman LM. 2009. Implications of changing climate for global wildland fire. Int J Wildland Fire 18:483-507.

Franzi LM, Bratt JM, Williams KM, Last JA. 2011. Why is particulate matter produced by wildfires toxic to lung macrophages? Toxicol Appl Pharmacol 257:182-188.

Gehring U, Tamburic L, Sbihi H, Davies HW, Brauer M. 2014. Impact of noise and air pollution on pregnancy outcomes. Epidemiology 25:351-358.

Gillett NP, Weaver AJ, Zwiers FW, Flannigan MD. 2004. Detecting the effect of climate change on Canadian forest fires. Geophysical Research Letters 31. doi:10.1029/2004GL020876

Haikerwal A, Akram M, Del Monaco A, Smith K, Sim MR, Meyer M, et al. 2015. Impact of fine particulate matter ($PM_{2.5}$) exposure during wildfires on cardiovascular health outcomes. J Am Heart Assoc 4:e001653 doi:10.1161/JAHA.114.001653.

Hanigan IC, Johnston FH, Morgan GG. 2008. Vegetation fire smoke, indigenous status and cardio-respiratory hospital admissions in Darwin, Australia, 1996-2005: A time-series study. Environ Health 7:42.

Henderson SB, Brauer M, Macnab YC, Kennedy SM. 2011. Three measures of forest fire smoke exposure and their associations with respiratory and cardiovascular health outcomes in a population-based cohort. Environ Health Perspect 119:1266-1271.

Henderson SB, Johnston FH. 2012. Measures of forest fire smoke exposure and their associations with respiratory health outcomes. Curr Opin Allergy Clin Immunol 12:221-227.

Ho RC, Zhang MW, Ho CS, Pan F, Lu Y, Sharma VK. 2014. Impact of 2013 south Asian haze crisis: Study of physical and psychological symptoms and perceived dangerousness of pollution level. BMC Psychiatry 14:81.

Holstius DM, Reid CE, Jesdale BM, Morello-Frosch R. 2012. Birth weight following pregnancy during the 2003 southern California wildfires. Environ Health Perspect 120:1340-1345.

Ignotti E, Valente JG, Longo KM, Freitas SR, Hacon SD, Netto PA. 2010. Impact on human health of particulate matter emitted from burnings in the Brazilian Amazon region. Rev Saude Publica 44:121-130.

Jacobson LSV, Hacon S, Castro HA, Ignotti E, Artaxo P, Ponce de Leon AC. 2012. Association between fine particulate matter and the peak expiratory flow of schoolchildren in the Brazilian subequatorial amazon: A panel study. Environ Res 117:27-35.

Jacobson LSV, Hacon Sde S, Castro HA, Ignotti E, Artaxo P, Saldiva PH, et al. 2014. Acute effects of particulate matter and black carbon from seasonal fires on peak expiratory flow of schoolchildren in the Brazilian Amazon. PloS One 9:e104177.

Jalaludin B, Smith M, O'Toole B, Leeder S. 2000. Acute effects of bushfires on peak expiratory flow rates in children with wheeze: A time series analysis. Aust N Z J Public Health 24:174-177.

Jayachandran S. 2009. Air quality and early-life mortality evidence from Indonesia's wildfires. J Hum Resour 44:916-954.

Johnston FH, Bailie RS, Pilotto LS, Hanigan IC. 2007. Ambient biomass smoke and cardio-respiratory hospital admissions in Darwin, Australia. BMC Public Health 7:240.

Johnston F, Hanigan I, Henderson S, Morgan G, Bowman D. 2011. Extreme air pollution events from bushfires and dust storms and their association with mortality in Sydney, Australia 1994-2007. Environ Res 111:811-816.

Johnston FH, Henderson SB, Chen Y, Randerson JT, Marlier M, Defries RS, et al. 2012. Estimated global mortality attributable to smoke from landscape fires. Environ Health Perspect 120:695-701.

Johnston FH, Kavanagh AM, Bowman D, Scott RK. 2002. Exposure to bushfire smoke and asthma: An ecological study. Med J Aust 176:535-538.

Johnston FH, Purdie S, Jalaludin B, Martin KL, Henderson SB, Morgan GG. 2014. Air pollution events from forest fires and emergency department attendances in Sydney, Australia 1996-2007: A case-crossover analysis. Environ Health 13:105.

Johnston FH, Webby RJ, Pilotto LS, Bailie RS, Parry DL, Halpin SJ. 2006. Vegetation fires, particulate air pollution and asthma: A panel study in the Australian monsoon tropics. Int J Environ Health Res 16:391-404.

Kong K, Coates HL. 2009. Natural history, definitions, risk factors and burden of otitis media. Med J Aust 191:S39-43.

Lakshmi PV, Virdi NK, Sharma A, Tripathy JP, Smith KR, Bates MN, et al. 2013. Household air pollution and stillbirths in India: Analysis of the dlhs-ii national survey. Environ Res 121:17-22.

Lee TS, Falter K, Meyer P, Mott J, Gwynn C. 2009. Risk factors associated with clinic visits during the 1999 forest fires near the Hoopa Valley Indian Reservation, California, USA. Int J Environ Health Res 19:315-327.

Leonard SS, Castranova V, Chen BT, Schwegler-Berry D, Hoover M, Piacitelli C, et al. 2007. Particle size-dependent radical generation from wildland fire smoke. Toxicology 236:103-113.

Linares C, Carmona R, Tobias A, Miron IJ, Diaz J. 2014. Influence of advections of particulate matter from biomass combustion on specific-cause mortality in Madrid in the period 2004-2009. Environ Sci Pollut Res. doi:10.1007/s11356-014-3916-2.

Liu JC, Pereira G, Uhl SA, Bravo MA, Bell ML. 2014. A systematic review of the physical health impacts from non-occupational exposure to wildfire smoke. Environ Res 136c:120-132.

MacIntyre EA, Karr CJ, Koehoorn M, Demers PA, Tamburic L, Lencar C, et al. 2011. Residential air pollution and otitis media during the first two years of life. Epidemiology 22:81-89.

Marshall GN, Schell TL, Elliott MN, Rayburn NR, Jaycox LH. 2007. Psychiatric disorders among adults seeking emergency disaster assistance after a wildland-urban interface fire. Psychiatr Serv 58:509-514.

Martin KL, Hanigan IC, Morgan GG, Henderson SB, Johnston FH. 2013. Air pollution from bushfires and their association with hospital admissions in Sydney, Newcastle and Wollongong, Australia 1994-2007. Aust NZ J Public Health 37:238-243.

Mazzoli-Rocha F, Magalhaes CB, Malm O, Saldiva PH, Zin WA, Faffe DS. 2008. Comparative respiratory toxicity of particles produced by traffic and sugar cane burning. Environ Res 108:35-41.

McDermott BM, Lee EM, Judd M, Gibbon P. 2005. Posttraumatic stress disorder and general psychopathology in children and adolescents following a wildfire disaster. Can J Psychiatry 50:137-143.

Miller LA, Schelegle ES, Capitanio JP, Clay CC, Walby WF. 2013. Persistent immune effects of wildfire PM exposure during childhood development. California Air Resources Board Contract Number 10-303

Moore D, Copes R, Fisk R, Joy R, Chan K, Brauer M. 2006. Population health effects of air quality changes due to forest fires in British Columbia in 2003: Estimates from physician-visit billing data. Can J Public Health 97:105-108.

Morgan G, Sheppeard V, Khalaj B, Ayyar A, Lincoln D, Jalaludin B, et al. 2010. Effects of bushfire smoke on daily mortality and hospital admissions in Sydney, Australia. Epidemiology 21:47-55.

Mott JA, Mannino DM, Alverson CJ, Kiyu A, Hashim J, Lee T, et al. 2005. Cardiorespiratory hospitalizations associated with smoke exposure during the 1997 southeast Asian forest fires. Int J Hyg Environ Health 208:75-85.

Mott JA, Meyer P, Mannino D, Redd SC, Smith EM, Gotway-Crawford C, et al. 2002. Wildland forest fire smoke: Health effects and intervention evaluation, Hoopa, California, 1999. West J Med 176:157-162.

Myatt TA, Vincent MS, Kobzik L, Naeher LP, MacIntosh DL, Suh H. 2011. Markers of inflammation in alveolar cells exposed to fine particulate matter from prescribed fires and urban air. J Occup Environ Med 53:1110-1114.

Naeher LP, Brauer M, Lipsett M, Zelikoff JT, Simpson CD, Koenig JQ, et al. 2007. Woodsmoke health effects: A review. Inhal Toxicol 19:67-106.

Nakayama Wong LS, Aung HH, Lame MW, Wegesser TC, Wilson DW. 2011. Fine particulate matter from urban ambient and wildfire sources from California's San Joaquin Valley initiate differential inflammatory, oxidative stress, and xenobiotic responses in human bronchial epithelial cells. Toxicol In Vitro 25:1895-1905.

Nunes KV, Ignotti E, Hacon Sde S. 2013. Circulatory disease mortality rates in the elderly and exposure to $PM_{2.5}$ generated by biomass burning in the Brazilian amazon in 2005. Cad Saude Publica 29:589-598.

Papanikolaou V, Adamis D, Mellon RC, Prodromitis G. 2011. Psychological distress following wildfires disaster in a rural part of Greece: A case-control population-based study. Int J Emerg Ment Health 13:11-26.

Pavagadhi S, Betha R, Venkatesan S, Balasubramanian R, Hande MP. 2013. Physicochemical and toxicological characteristics of urban aerosols during a recent Indonesian biomass burning episode. Environ Sci Pollut Res Int 20:2569-2578.

Prass TS, Lopes SR, Dorea JG, Marques RC, Brandao KG. 2012. Amazon forest fires between 2001 and 2006 and birth weight in Porto Velho. Bull Environ Contam Toxicol 89:1-7.

Rappold AG, Cascio WE, Kilaru VJ, Stone SL, Neas LM, Devlin RB, et al. 2012. Cardio-respiratory outcomes associated with exposure to wildfire smoke are modified by measures of community health. Environ Health 11:71.

Rappold AG, Stone SL, Cascio WE, Neas LM, Kilaru VJ, Carraway MS, et al. 2011. Peat bog wildfire smoke exposure in rural North Carolina is associated with cardiopulmonary emergency department visits assessed through syndromic surveillance. Environ Health Perspect 119:1415-1420.

Resnick A, Woods B, Krapfl H, Toth B. 2015. Health outcomes associated with smoke exposure in Albuquerque, New Mexico, during the 2011 wallow fire. J Public Health Manag Pract. 21 Suppl 2:S55-61.

Sastry N. 2002. Forest fires, air pollution, and mortality in southeast asia. Demography 39:1-23.

Settele J, Scholes R, Betts R, Bunn S, Leadley P, Nepstad D, Overpeck JT, Taboada MA, 2014: Terrestrial and inland water systems. In: *Climate Change 2014: Impacts, Adaptation, and Vulnerability. Part A: Global and Sectoral Aspects. Contribution of Working Group II to the Fifth Assessment Report of the Intergovernmental Panel on Climate Change* [Field, C.B., V.R. Barros, D.J. Dokken, K.J. Mach, M.D. Mastrandrea, T.E. Bilir, M. Chatterjee, K.L. Ebi, Y.O. Estrada, R.C. Genova, B. Girma, E.S. Kissel, A.N. Levy, S. MacCracken, P.R. Mastrandrea, L.L. White (eds.)]. Cambridge University Press, Cambridge, United Kingdom and New York, NY, USA, pp. 271-359.

Shaposhnikov D, Revich B, Bellander T, Bedada GB, Bottai M, Kharkova T, et al. 2014. Mortality related to air pollution with the Moscow heat wave and wildfire of 2010. Epidemiology 25:359-364.

Silveira HC, Schmidt-Carrijo M, Seidel EH, Scapulatempo-Neto C, Longatto-Filho A, Carvalho AL, et al. 2013. Emissions generated by sugarcane burning promote genotoxicity in rural workers: A case study in Barretos, Brazil. Environ Health 12:87.

Sisenando HA, Batistuzzo de Medeiros SR, Artaxo P, Saldiva PH, Hacon Sde S. 2012. Micronucleus frequency in children exposed to biomass burning in the Brazilian legal amazon region: A control case study. BMC Oral Health 12:6.

Smith MA, Jalaludin B, Byles JE, Lim L, Leeder SR. 1996. Asthma presentations to emergency departments in western Sydney during the January 1994 bushfires. Int J Epidemiol 25:1227-1236.

Tan WC, Qiu DW, Liam BL, Ng TP, Lee SH, van Eeden SF, et al. 2000. The human bone marrow response to acute air pollution caused by forest fires. Am J Respir Crit Care Med 161:1213-1217.

Tham R, Erbas B, Akram M, Dennekamp M, Abramson MJ. 2009. The impact of smoke on respiratory hospital outcomes during the 2002-2003 bushfire season, Victoria, Australia. Respirology 14:69-75.

Thelen B, French NH, Koziol BW, Billmire M, Owen RC, Johnson J, et al. 2013. Modeling acute respiratory illness during the 2007 San Diego wildland fires using a coupled emissions-transport system and generalized additive modeling. Environ Health 12:94.

Tse K, Chen L, Tse M, Zuraw B, Christiansen S. 2015. Effect of catastrophic wildfires on asthmatic outcomes in obese children: Breathing fire. Ann Allergy Asthma Immunol 114:308-311 e304.

van Eeden SF, Tan WC, Suwa T, Mukae H, Terashima T, Fujii T, et al. 2001. Cytokines involved in the systemic inflammatory response induced by exposure to particulate matter air pollutants (PM_{10}). Am J Respir Crit Care Med 164:826-830.

Vedal S, Dutton SJ. 2006. Wildfire air pollution and daily mortality in a large urban area. Environ Res 102:29-35.

Vora C, Renvall MJ, Chao P, Ferguson P, Ramsdell JW. 2011. 2007 San Diego wildfires and asthmatics. Journal Asthma 48:75-78.

Wegesser TC, Franzi LM, Mitloehner FM, Eiguren-Fernandez A, Last JA. 2010. Lung antioxidant and cytokine responses to coarse and fine particulate matter from the great California wildfires of 2008. Inhal Toxicol 22:561-570.

Wegesser TC, Pinkerton KE, Last JA. 2009. California wildfires of 2008: Coarse and fine particulate matter toxicity. Environ Health Perspect 117:893-897.

Westerling AL, Hidalgo HG, Cayan DR, Swetnam TW. 2006. Warming and earlier spring increase western us forest wildfire activity. Science 313:940-943.

Williams KM, Franzi LM, Last JA. 2013. Cell-specific oxidative stress and cytotoxicity after wildfire coarse particulate matter instillation into mouse lung. Toxicol Appl Pharmacol 266:48-55.

Wiwatanadate P, Liwsrisakun C. 2011. Acute effects of air pollution on peak expiratory flow rates and symptoms among asthmatic patients in Chiang Mai, Thailand. Int J Hyg Environ Health 214:251-257.

Woodruff TJ, Parker JD, Adams K, Bell ML, Gehring U, Glinianaia S, et al. 2010. International collaboration on air pollution and pregnancy outcomes (ICAPPO). Int J Environ Res Public Health 7:2638-2652.

Woodruff TJ, Sutton P. 2014. The navigation guide systematic review methodology: A rigorous and transparent method for translating environmental health science into better health outcomes. Environ Health Perspect 122:1007-1014.

Yao J, Eyamie J, Henderson SB. 2014. Evaluation of a spatially resolved forest fire smoke model for population-based epidemiologic exposure assessment. J Expo Sci Environ Epidemiol. doi:10.1038/jes.2014.67

Youssouf H, Liousse C, Roblou L, Assamoi EM, Salonen RO, Maesano C, et al. 2014. Non-accidental health impacts of wildfire smoke. Int J Environ Res Public Health 11:11772-11804.

Zelikoff JT, Chen LC, Cohen MD, Schlesinger RB. 2002. The toxicology of inhaled woodsmoke. J Toxicol Environ Health B Crit Rev 5:269-282.
